# Accuracy of diagnostic tests in cardiac injury after blunt chest trauma: a systematic review and meta-analysis

**DOI:** 10.1186/s13017-023-00504-9

**Published:** 2023-05-27

**Authors:** Ioannis Panagiotis Kyriazidis, Dominik A. Jakob, Juliana Alexandra Hernández Vargas, Oscar H. Franco, Elias Degiannis, Patrick Dorn, Sjaak Pouwels, Bijendra Patel, Ian Johnson, Christopher John Houdlen, Graham S. Whiteley, Marion Head, Anil Lala, Haroon Mumtaz, J. Agustin Soler, Katie Mellor, David Rawaf, Ahmed R. Ahmed, Suhaib J. S. Ahmad, Aristomenis Exadaktylos

**Affiliations:** 1 Department of Emergency Medicine, Inselspital University Hospital of Bern, Bern, Switzerland; 2 Department of Global Public Health and Bioethics, Julius Center for Health Sciences and Primary Care, University Medical Center (UMC) Utrecht, Utrecht, The Netherlands; 3 Department of Surgery, University of Witwatersrand Medical School, Johannesburg, South Africa; 4 Department of Thoracic Surgery, Inselspital, University Hospital of Bern, Bern, Switzerland; 5 Department of General, Abdominal and Minimally Invasive Surgery, Helios Klinikum Krefeld, Krefeld, Germany; 6 Department of General Surgery, Barts Cancer Institute, London, UK; 7 Department of Anaesthesia & Intensive Care, Betsi Cadwaladr University Health Board, Bodelwyddan, Wales, UK; 8 Department of General Surgery, Betsi Cadwaladr University Health Board, Bangor, LL57 2PW Wales, UK; 9 Department of Trauma and Orthopaedic Surgery, Betsi Cadwaladr University Health Board, Bangor, Wales, UK; 10 Department of Surgery, South West London Orthopaedic Centre, London, UK; 11 Department of General Surgery, Imperial College London, London, UK

**Keywords:** Blunt chest trauma, Cardiac contusion, Cardiac concussion, Commotio cordis, Contusio cordis, Diagnostic protocol

## Abstract

**Introduction:**

The diagnosis of cardiac contusion, caused by blunt chest trauma, remains a challenge due to the non-specific symptoms it causes and the lack of ideal tests to diagnose myocardial damage. A cardiac contusion can be life-threatening if not diagnosed and treated promptly. Several diagnostic tests have been used to evaluate the risk of cardiac complications, but the challenge of identifying patients with contusions nevertheless remains.

**Aim of the study:**

To evaluate the accuracy of diagnostic tests for detecting blunt cardiac injury (BCI) and its complications, in patients with severe chest injuries, who are assessed in an emergency department or by any front-line emergency physician.

**Methods:**

A targeted search strategy was performed using Ovid MEDLINE and Embase databases from 1993 up to October 2022. Data on at least one of the following diagnostic tests: electrocardiogram (ECG), serum creatinine phosphokinase-MB level (CPK-MB), echocardiography (Echo), Cardiac troponin I (cTnI) or Cardiac troponin T (cTnT). Diagnostic tests for cardiac contusion were evaluated for their accuracy in meta-analysis. Heterogeneity was assessed using the *I*^2^ and the QUADAS-2 tool was used to assess bias of the studies.

**Results:**

This systematic review yielded 51 studies (n = 5,359). The weighted mean incidence of myocardial injuries after sustaining a blunt force trauma stood at 18.3% of cases. Overall weighted mean mortality among patients with blunt cardiac injury was 7.6% (1.4–36.4%). Initial ECG, cTnI, cTnT and transthoracic echocardiography TTE all showed high specificity (> 80%), but lower sensitivity (< 70%). TEE had a specificity of 72.1% (range 35.8–98.2%) and sensitivity of 86.7% (range 40–99.2%) in diagnosing cardiac contusion. CK-MB had the lowest diagnostic odds ratio of 3.598 (95% CI: 1.832–7.068). Normal ECG accompanied by normal cTnI showed a high sensitivity of 85% in ruling out cardiac injuries.

**Conclusion:**

Emergency physicians face great challenges in diagnosing cardiac injuries in patients following blunt trauma. In the majority of cases, joint use of ECG and cTnI was a pragmatic and cost-effective approach to rule out cardiac injuries. In addition, TEE may be highly accurate in identifying cardiac injuries in suspected cases.

**Supplementary Information:**

The online version contains supplementary material available at 10.1186/s13017-023-00504-9.

## Introduction

Blunt chest injuries (BCI) account for 15% of admissions to emergency departments worldwide and they are associated with significant morbidity and mortality (1–3). They may occur following road collision accidents, falls from heights, physical assaults or athletic injuries. The injury sustained to the heart is thought to be due to decelerating forces on the anterior aspect of the chest wall, which challenges the viscoelastic properties of the heart [1]. After the impact, the heart can move freely within the thoracic cavity on the anterior–posterior axis and may then either be solely compressed against the posterior aspect of the sternum or—after a more energetic impact—be “squashed” between the sternum and the anterior aspect of the thoracic spine.

As blunt chest injuries encompass an entire spectrum of different mechanisms and intensities, it is evident that the clinical presentation of these patients varies greatly. Presentations range from a silent clinical picture to differing degrees of physiological instability, but may in certain cases be “catastrophic” if important structures have been injured—such as the pericardium, the valves, the papillary muscle, chordae tendinae, the ventricular septum, or coronary artery [2–4].

In the literature, the terms BCI and cardiac contusion are often used loosely. To clarify and correct this issue, a consensus statement published in the Journal of Trauma, by Mattox et al., stated that the term cardiac contusion should cease to be used as a diagnosis for admission or scoring of injury severity [5]. They proposed a nomenclature of BCI for all cardiac injuries and accepted that the term “cardiac contusion” can also be used in more benign cases. Therefore, the term BCI covers blunt cardiac injury with septal rupture, free wall rupture, coronary artery thrombosis, cardiac failure, minor ECG or enzyme abnormalities alongside cardiac arrhythmias, with the last three also being termed “myocardial contusion”.

In the emergency department, BCI presents in three clinical groups:

- In the presence of septal rupture, and/or free wall rupture, and/or coronary artery thrombosis, the patient usually dies on site. In the emergency department, the patient is physiologically unstable, and a swift diagnosis will be made—followed by transfer to the operating theatre or admission to an intensive care unit.
- In the “myocardial contusion” group, a high threshold of suspicion coupled with investigations is often required, as the clinical picture can be obscured by concomitant injuries.
- Patients involved in accidents with severe blunt injury to the chest may have sustained BCI without any significant clinical symptoms or signs [5] [6].

Therefore, every patient with severe blunt trauma to the chest will have to be investigated for the presence of BCI, so a decision can then be taken to discharge or admit them to the hospital [7].

The most frequently used investigations are electrocardiography (ECG), echocardiography (Echo), cardiac biomarkers and radioisotope scanning [8]. If the initial 12–lead ECG is normal, it is recommended to take the 4–6 h ECG, while parallel measurement of cardiac biomarkers such as troponin and creatine kinase (CK) is recommended [7–11]. Follow-up measurements of cardiac biomarkers and additional Echo or radioisotope scans depend on clinical findings, the ECG and the measurement of cardiac biomarkers.

However, the management of these patients is highly variable and depends on hospital protocols [12].

The diagnostic procedure for BCI and its associated complications in trauma patients remains challenging. This meta-analysis aims to provide an evidence-based starting point in reaching a consensus for diagnosing BCI in the setting of the emergency department.

## Methods

**Fig. 1:**
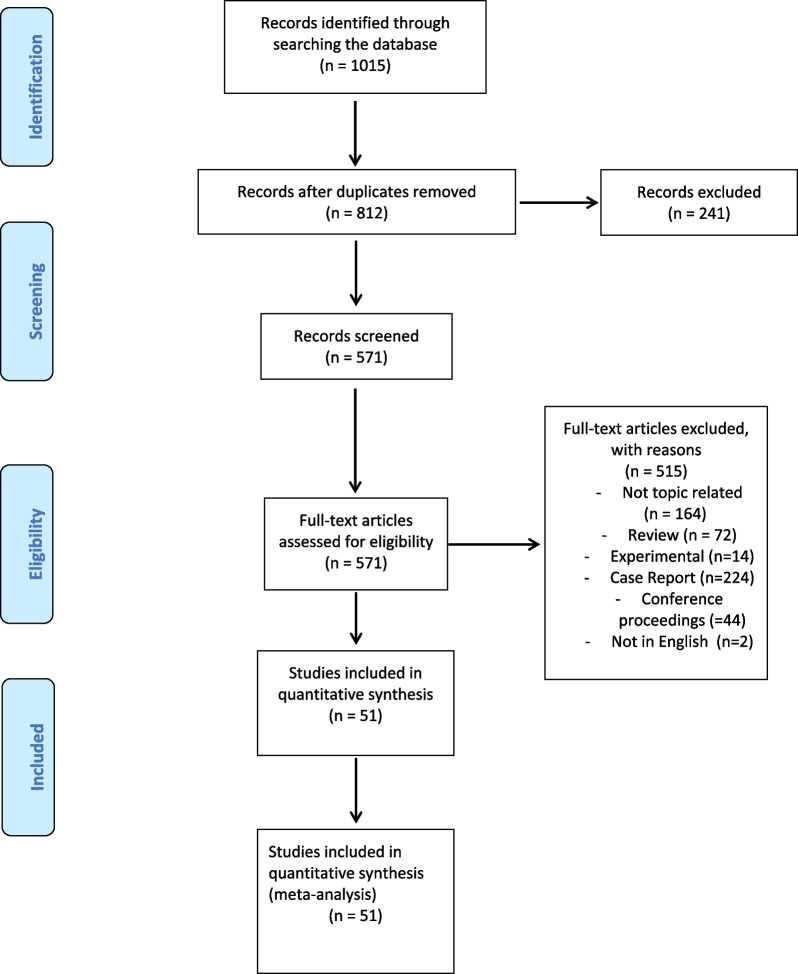
PRISMA flowchart

This systematic review and meta-analysis was reported in accordance with the Preferred Reporting Items for Systematic Reviews and Meta-analysis (PRISMA) 2020 guidelines [13] (Fig. 1).

### Primary objectives

The primary aim of this study is to evaluate the accuracy of electrocardiogram (ECG), serum creatinine phosphokinase-MB level (CPK-MB), transoesophageal echocardiography (TEE), transthoracic echocardiography (TTE), cardiac troponin T (cTnT) and cardiac troponin I (cTnI) in the diagnosis of blunt cardiac injury, and its complications.

### Secondary objectives

The secondary objective of this study was to establish a propagated algorithm for the initial assessment of cardiac contusion, in patients with a blunt chest injury.

### Databases and search strategy

Two independent reviewers searched Ovid MEDLINE and Embase databases to identify relevant articles from 1993 to October 12, 2022. The search was restricted to articles published in English. Articles were then screened by reading the title, abstract and full text. The detailed search strategy is listed in Additional file 1: Table S1.

### Inclusion criteria

Articles were considered if they fulfilled all the following criteria:

- They were original articles (cohort studies, case-controlled studies, case series, cross-sectional studies)
- They contained details about patients with blunt chest trauma, who were suspected of suffering myocardial contusion.
- They included data on at least one of the following: ECG, CPK-MB, TEE, TTE, cTnT or cTnI.

### Exclusion criteria

- Review articles (systematic and narrative reviews)
- Case reports
- Experimental articles
- Penetrating thoracic trauma
- FAST and e-FAST scans
- CT scans
- MRI scans

### Definition of an abnormal test

ECG: Any new abnormality in conduction, rhythm or rate that was thought to be consistent with cardiac injury.
CPK-MB: Generally accepted as above the normal threshold.cTnI: Generally accepted as above the normal threshold.cTnT: Generally accepted as above the normal threshold.
TTE: Any detected abnormality in function or structure that is consistent with cardiac injury. This included dyskinesia/akinesia, anatomical disruption or echo-dense areas in the myocardium.
TEE: Any detected abnormality in function or structure that is consistent with cardiac injury. This included dyskinesia/akinesia, anatomical disruption or echo-dense areas in the myocardium.

### Definition of a complication

A complication was defined as any new cardiac problem. Non-sustained ventricular tachycardia, premature ventricular contractions (PVCs) and transient bundle branch block were regarded as complications. Patients with pre-existing cardiac conditions were excluded from the study unless they required additional treatment.

### Definition of cardiac injuries

- Autopsy-proven contusion, or laceration of the myocardium.
- Contusion injury of the myocardium, as proven in Echo, CT MRI scans.
- Free wall rupture leading to cardiac tamponade, haemorrhage, dyskinesia, pericardial laceration, and effusion.
- Coronary artery thrombosis.
- Congestive heart failure (CCF) following injury.
- Serious arrhythmias include supraventricular tachycardia (SVT), ventricular tachycardia (VT), atrial fibrillation (AF), and new Premature Ventricular Complex (PVC)). New conduction blocks were included with. new onset significant ST changes; non-specific ST changes were not included.
- Radionuclide/multi-gated acquisition scan (MUGA)/single-photon emission computerized tomography (SPECT) scan showing a new change in cardiac structure or activity.

### Quality assessment

Articles were assessed independently by three epidemiologically trained investigators. Disputes were resolved by consensus. The Oxford Centre for the Level of Evidence and the quality assessment with diverse studies (QUADAS-2) was utilised to assess the quality and the risk of bias of the articles included. This is regarded as a valid tool to assess the methodological bias and the applicability [14]. Heterogeneity was assessed using the I^2^.

### Outcomes measures

Article title, first author, first author’s country, year of publication, journal, journal quartile, journal country of origin, article type (randomised controlled trials, cohort studies, case–control, case-series), article classification (prospective/retrospective), level of evidence (I-V), number of patients in the study, number of cardiac injuries in the study, age of participants, gender of participants, cardiac injury (serious arrhythmias, benign arrhythmias, conduction abnormalities, repolarisation abnormalities, ST/T/Q wave changes, dyskinesia/insufficiency/structural changes, pericarditis, effusion/thrombus, tamponade, deaths and its causes including cardiac rupture, haemorrhage/effusion/contusion, dyskinesia/hypokinesia, refractory arrhythmias, non-cardiac causes, hypotension, infarct/thrombus, tamponade and CCF.

### Statistical analysis

The descriptive and meta-analysis were performed using OpenMeta of OpenMetaAnalyser (Version R01 HS 018574) and IBM SPSS Statistics Version 29.0.0.0 (241). The ordinary arithmetic mean (weighted mean) was used to calculate theoretically expected outcomes, where each outcome has a different probability of occurring. Weighted mean = Σ(w)_n_ (x̄)_n_/Σ(w)_n_. The diagnostic accuracy of a test is classically estimated by its sensitivity and specificity. However, these two elements of test performance have a bivariate relationship, meaning that they are linked through the choice of the threshold at which a test is considered ‘positive’ or ‘negative’. Thus a receiver operating characteristics (ROC) curve meta-analysis (which takes into account the paired nature of sensitivity and specificity) was created. For this analysis, we used the methodology proposed by Kester et al. [15].

### Statistical outcomes

- Diagnostic odds ratio (DOR)
- Negative likelihood ratio (NLR)
- Positive likelihood ratio (PLR)
- Sensitivity
- Specificity

## Results

Fifty-one (51) studies were included in this meta-analysis. The search strategy is summarised in Additional file 1: Table S1 and the PRISMA flowchart is described in Fig. 1. The QUADAS-2 tool results are demonstrated in Additional file 1: Table S2.

The methodological quality of the studies:

### Applicability

35 out of the 51 studies exhibited a low-risk patient selection and the remaining 16 exhibited an unclear risk. All 51 studies exhibited low-risk index testing and reference standards.

### Bias

34 out of the 51 studies exhibited a low-risk patient selection. All 51 studies exhibited low-risk index testing and reference standards. 44 out of the 51 studies exhibited a low-risk flow and timing, whilst 7 exhibited an unclear risk.

### Study characteristics

The characteristics of the included 51 articles reporting on 5,359 patients are shown in Additional file 1: Table S3. The median (range) number of patients per article was 66 (3–993).

### Articles

Twenty-five (25) of the studies were prospective and 26 articles were retrospective. Seven studies were case–control, 42 were case series and two were cohort studies. 49 articles were of level IV and 2 of level II. Most of the articles were published in 1996 (5), followed by 2021(5), 1995(4), 2001(4), 1997(3), 2002(3) and 2017(3).

### Cardiac complications in patients with cardiac contusion

The incidence of cardiac injury, in patients with blunt chest trauma stood at 18.32%.

### ECG and Echo changes

The weighted mean risk of serious arrhythmias (AF, PVC, VT, SVT) in patients with blunt cardiac injury stood at 20.7% (14.6–24.3%). The weighted mean risk of experiencing benign arrhythmias stood at 26.3% (25–33.3%). The weighted mean conduction abnormalities, amongst patients with cardiac injury, stood at 24.6% (14.3–75%). A weighted mean of 33.3% of patients had repolarisation abnormalities. A weighted mean of 45.9% (12.5–100%) of patients experienced ST, T or Q wave changes and these included both specific and non-specific changes. A weighted mean of 30.7% (15.7–100%) of patients with cardiac injury had dyskinesis, insufficiency or structural changes. A weighted mean of 15.2% (12.5–100%) of the patients had effusion or a thrombus. Cardiac tamponade was detected in 1.4%.

### Mortality in patients with cardiac injury

The weighted mean mortality rate amongst patients who sustained a cardiac injury stood at 7.6% (1.4–36.4%). A weighted mean of 8.6% (8–10%) of deceased patients exhibited cardiac rupture. A weighted mean of 6.9% (4–25%) of deceased patients exhibited haemorrhage, effusion, or contusion. Dyskinesia was noted in 10% of the deceased. A weighted mean of 84.6% (4–25%) of patients with cardiac contusion died from non-cardiac-related causes. Cardiac failure was noted in 100% of deaths.

### Diagnostic accuracy of different tests for cardiac injury in blunt chest trauma

Table 1 summarises the diagnostic accuracy of different tests for blunt cardiac injury. Initial ECG, cTnI, cTnT, and TTE all exhibited high specificity (> 80%) but lower sensitivity (< 70%). TEE had the highest specificity of 72.1% (range 35.8–98.2%) and sensitivity of 86.7% (range 40–99.2%) in diagnosing cardiac contusion. CK-MB had the lowest diagnostic odds ratio of 3.598 (95% CI: 1.832–7.068). A normal ECG in combination with a normal cTnI was excellent in ruling out blunt cardiac injury (sensitivity of 85%). Detailed information on the diagnostic accuracy of the various tests is provided in Additional file 1: Table S4-S9b and Additional file 2–26: Figs. S1–S25. The ROC curves are presented in Additional file 27–30: Figs. S26–S29.

**Table 1:** Diagnostic accuracy of different tests and test combinations for the diagnosis of blunt cardiac injury

	Total number of studies N = 51					
	Sensitivity	Specificity	PLR	NLR	DOR	Studies
Electrocardiography (ECG)	55.1% (95% CI: 45.2–64.6)	84.5 (95% CI: 74.6–91.0)	2.719 (95% CI: 1.654–4.469)	0.494 (95% CI: 0.309–0.789)	5.975 (95% CI: 2.675–13.349)	28
Troponin I	64.4% (95% CI: 52.3–74.9)	84.1% (95% CI: 72.3–91.5)	3.792 (95% CI: 2.181–6.594)	0.329 (95% CI: 0.170–0.638)	15.009 (95% CI: 6.910–32.603)	17
Troponin T	68.4% (95% CI: 40.2–87.5)	85.8% (95% CI: 73.6–92.9)	4.368 (95% CI: 2.473–7.715)	0.217 (95% CI: 0.120–0.395)	16.048 (95% CI: 7.453—34.558)	6
Creatine phosphokinase-MB (CK-MB)	55.2% (95% CI: 43.1–66.6)	75.8% (95% CI: 62.7–85.4)	1.927 (95% CI: 1.370–2.711)	0.523 (95% CI: 0.386–0.710)	3.598 (95% CI: 1.832–7.068)	22
Transthoracic Echocardiography (TTE)	47.0% (95% CI: 34.2–60.2)	91.4 (95% CI: 84.3–95.5)	3.558 (95% CI: 1.967–6.436)	0.446 (95% CI: 0.249–0.799)	10.077 (95% CI: 3.845–26.408)	20
Transoesophageal Echocardiography (TEE) (case reports included)	93.1% (95% CI: 76.5–98.2)	87.6% (95% CI: 46.1–98.3)	5.711 (95% CI: 1.065–30.613)	0.131 (95% CI: 0.034–0.513)	59.994 (95% CI: 1.947–1848.814)	5
Transoesophageal Echocardiography (TEE) (case reports excluded)	Sensitivity: 86.7% (range 40.0–99.2)	Specificity: 72.1% (range 35.8–98.2)	NA	NA	NA	3
Electrocardiography (ECG) and Troponin I*	34%	75%	NA	NA	NA	10
Electrocardiography (ECG) or Troponin I*	87.5%	25%	NA	NA	NA	11

*PLR* positive likelihood ratio; *NLR* negative likelihood ratio, *DOR* diagnostic odds ratio

*No confidence interval was reported for the ECG and Troponin test combination

### Articles and journals

The 51 articles were published in 34 journals. 11 out of the 34 journals originated from the USA, 5 originated from the UK, 5 from Turkey, 2 from Germany, 2 from India and 2 from the Netherlands. 23 out of the 51 articles originated from the USA, 6 articles originated from the UK and 5 from Turkey and 5 from The Netherlands. Germany and India contributed three and two articles respectively. Taiwan, Singapore, Italy, Ireland, Egypt, Canada and Austria contributed each 1 article (Additional file 1: Table S10).

### Authors

A R Edouard was the only author to publish 2 articles. Most of the first authors originated from 18 countries, including the USA (13), Turkey (5), Germany (5), France (5), the Netherlands (3), Taiwan (3), Switzerland (2), UK (2), Iran (2), Israel (2), Italy (2), Austria (1), Belgium (1), Canada (1), Greece (1), India (1), Malaysia (1), Qatar (1). See Additional file 1: Table S11.

### Patient data

The weighted mean blunt cardiac injury rate amongst admitted patients was 18.3% (0–100%). The weighted mean age for all patients was 38.7 (0–55.85) years.

The weighted mean age of patients who had cardiac injury was 24.6 (8.2–52) years.

Amongst patients with blunt cardiac injury, 73.3% were males.

## Discussion

The diagnosis of blunt cardiac injury is challenging, as patients may present with non-specific symptoms and there is no golden diagnostic test. We report the incidence of cardiac injury in patients with blunt chest injuries to be 18.32%, which is consistent with the wide range reported in the literature, ranging between 3 and 56%, depending on the diagnostic criteria [16–20]. Furthermore, our systematic review revealed a weighted mean mortality rate for patients presenting with BCI of around 7.6%. Mortality rates for BCI vary in the existing literature. This can be explained by different study populations and the method used to detect BCI. Patients with cardiac injuries that result in immediate death are generally not included in studies [21–23]. This suggests that cardiac injuries are more common than widely reported. In line with these considerations, an autopsy study examining 881 cadavers with blunt trauma found a cardiac injury rate of 32%. According to the diagnostic challenges in BCI, we identified the use of ECG in combination with cTnI as a pragmatic approach to rule out cardiac injuries. In addition, TEE with a high specificity and sensitivity may be highly accurate in identifying cardiac injuries in suspected cases.

It is worth noting that severe cardiac injuries can easily be detected, as they usually present with haemodynamic instability. However, less severe injuries can easily be masked in trauma patients, due to other severe vascular, pulmonary, or neurological injuries. The biggest challenge revolves around diagnosing trauma patients with no clinical signs of myocardial injuries, as some present with only mild symptoms, such as palpitations or precordial pain, which are often related to the associated musculoskeletal injury. For instance, we reported that 20.7%, of the patients with blunt cardiac injury presented with serious arrhythmias (AF, PVC, VT, SVT). This leaves 79.3% of patients with a cardiac injury who presented with benign, less serious, arrhythmias.

The hypothesis states that cardiac injury, in patients with blunt chest trauma, is caused by deceleration forces affecting the viscoelastic properties of the chest wall leading to either direct pressure on the myocardium or an indirect pressure through increased intrathoracic pressure leading to shearing stresses. The histological findings seen in blunt cardiac injuries are similar to those seen in myocardial infarction, and these are intramyocardial haemorrhage, oedema and necrosis of cardiac cells [24, 25]. Thus, cardiac enzymes, such as troponin and creatinine kinase MB, were the first screening tools to detect cardiac injury. We reported that the sensitivity of CPK-MB was 55.2% and the specificity 75.8%. CPK-MB tends to have higher specificity in detecting acute myocardial infarction—but not in trauma patients with associated skeletal injuries [4, 24]. Thus, creatinine kinase is useful in detecting cardiac contusion in patients with none or only minor non-cardiac injuries. However, it is also worth noting that some authors have reported reduced sensitivity and specificity in patients with mild injuries [18, 25–28].

Our study has shown that cTnT has a sensitivity and specificity of 68.4% and 85.5%, respectively. The numbers for cTnI were 64.4% and 84.1%, respectively. The increased sensitivity and specificity compared to CPK-MB can be explained by the fact that both serum cardiac troponins are regulatory contractile proteins that are only present in heart muscle cells and not in skeletal muscles. In case of heart muscle damage resulting in loss of cell membrane integrity cardiac troponins are released into the serum. That makes troponin invaluable in diagnosing heart damage. This meta-analysis has also shown that cTnT has a negative likelihood ratio of 0.217% and a positive likelihood ratio of 4.368%. The values for cTnI were 0.329% and 3.792%, respectively. This led to the conclusion that positive serum cardiac troponins are accurate in the diagnosis of cardiac contusion. Furthermore, negative serum troponin is a strong indicator of the absence of the disease. The results are consistent with previous studies [18, 20, 29, 30]. The optimal time of sampling for serum troponin after trauma has not been established. However, it is proposed that if an initial serum troponin is negative, a second measurement should be performed after 4–6 h. High troponin levels tend to persist for 4–6 days, and this can aid with the diagnosis of blunt cardiac injury in late presentations [18, 31].

This study has shown that the ECG may be normal after a blunt chest injury or may show specific or non-specific abnormalities. Non-specific changes are also seen in trauma patients, some caused by anaemia, hypoxia, electrolyte abnormality and sympathetic and parasympathetic tone. This is in accordance with many other studies [18, 32]. ECG changes tend to reflect the activity of the left ventricle, as this is larger than the right ventricle and close to the sternum. Thus, contusions affecting the left ventricle can present as significant ST-changes and serious arrhythmias on the ECG, whilst, on the other hand, right ventricle contusion can easily be missed [33]. This study showed that the ECG has a sensitivity of 55.1% and a relatively good specificity of 84.5%. For instance, ST, T, or Q wave changes were present in 45.9% of patients with blunt cardiac injury. Furthermore, 20.7% of the patients with blunt cardiac injury presented with serious arrhythmias. This might be due to electrical instability, as these patients are usually more haemodynamically compromised and this finding is often associated with sudden death [19]. If we consider patients diagnosed with a blunt cardiac injury, 24.6% presented with conduction abnormalities. According to the latest literature, a right bundle branch block resembles a severely injured right ventricle. Other types of conduction abnormalities have also been reported in patients with cardiac contusion [16, 24, 32].

In light of our results, when both the ECG AND cTnI are positive the sensitivity of the diagnosis of blunt cardiac injury decreases. When only one of the ECG OR cTnI is positive, the sensitivity of the diagnosis of cardiac injury increases and the specificity decreases. Normal ECG in combination with a normal troponin I was excellent in ruling out blunt cardiac injury (sensitivity of 87.5%). In addition, the diagnostic combination of ECG and cTnI is widely available and offers an excellent cost-effectiveness ratio.

The effects of blunt cardiac injury are not only histological and may affect the function of the heart. On a 2D echo scan, BCI can be diagnosed as the presence of localised dysfunction of the myocardial wall [16]. An echo scan can also be utilised to detect valvular abnormalities, effusions, thrombus, and dilatations. However, it can be difficult to detect myocardial oedema without abnormal motion on a 2D echo [19]. We reported moderate sensitivity (47.0%) and high specificity (91.4%) for TTE. The findings for TEE were significantly higher—at 86.7% and 72.1%, respectively. In particular, posterior cardiac structures which are close to the oesophagus can be better visualized by TEE compared to TTE. A recently published review also confirmed that TEE can be performed at the bedside in different locations and also in mechanically ventilated patients [33].

Thus TEE is a good option to confirm or exclude BCI in suspected cases. In addition, it can also be used to detect suspected lesions in the great vessels. However, both Echo examinations have their limitations: TTE cannot be used in patients with severe chest wall injuries, as it can be very painful and TEE is contraindicated in patients with diagnosed or suspected oesophageal injury and should be used carefully in cervical spine injuries. In addition, Echo is also not as widely available as ECG or cardiac biomarkers. Furthermore, TEE requires advanced skills.

Although the transoesophageal investigation has been proven to be safe in patients with blunt chest trauma, the risk of adverse events is higher than with ECG or biomarker measurements. Iatrogenic injury may occur when the endoscope is inserted into the oesophagus, and sedation with all its associated risks is also necessary [34, 35]. A study published by OH JK reported breathing problems and haemodynamic instability in patients who underwent a TEE [36].

**Fig. 2:**
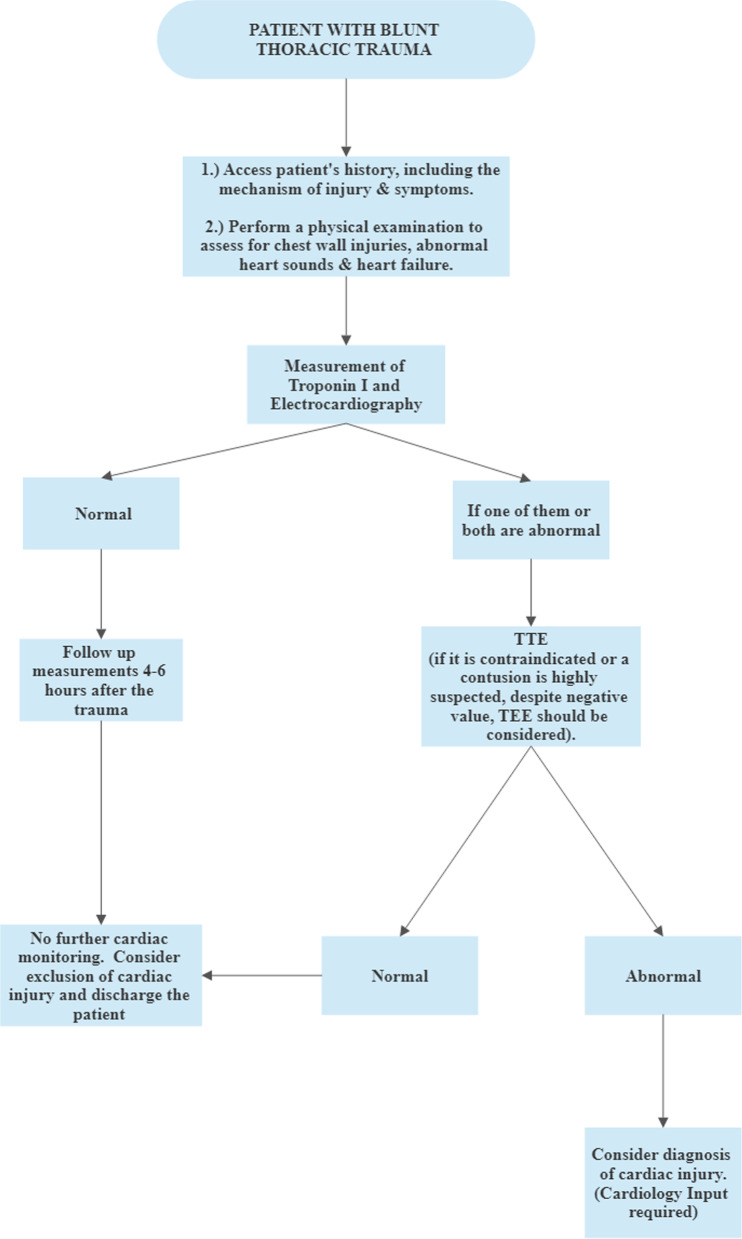
A propagated algorithm for the initial assessment of cardiac contusion

After weighing up the various advantages and disadvantages of each diagnostic test, we propose a simple algorithm for the initial assessment of cardiac injury in blunt chest trauma (Fig. 2).

### Who should perform the transthoracic echocardiography TTE and the transesophageal echocardiography TEE?

A TTE should be typically done by a cardiologist or a trained emergency physician. A TEE on the other side should be performed by a cardiologist. The exam should be interpreted by a physician trained in reading echocardiograms; ideally, that should be the cardiologist.

## Limitations

The certainty of a meta-analysis is dependent on the design and the level of evidence of the included studies. The sample size was simply not large enough for us to only include RCTs. Thus, this meta-analysis also included consecutive and non-consecutive case series. For the TEE, if we excluded case reports from the analysis, the number of included studies was small and this allowed the weighted mean to be calculated. For the reviewers interest, we have also included the values, of case reports were taken into consideration.

The quantitative analysis from such series were mainly used to estimate prevalence or event rates. All studies also underwent an independent quality check (QUDAS-2), to assess the methodology, ascertainment, causality and reporting. Inferences from such reports are commonly used for decision-making.

Publication bias was not assessed in this study. Furthermore, there was heterogeneity among the published studies. The ROC curves could not be calculated for TEE, due to the small number of studies. Moreover, the normal laboratory findings were not the same in different studies. This is due to various factors including instrument calibrations, reagents and controls used, the technique used, the sample used, and the ability of the person dealing with the sample to rectify the results. The choice of the cut-off point for a positive result affects the test accuracy. Thus this is regarded as our main statistical source of heterogeneity. To compensate for the heterogeneity, we considered a random effect model and we also created an ROC curve, which is a graphical curve, used to represent the performance of a test over a range of threshold settings. Thus allowing the analysis of sensitivity and specificity over a range of threshold settings. In addition, the time between the injury and the blood sampling time is not recorded in many studies, which can affect the blood results. An echo scan is also operator dependant and in most studies, it was not recorded who had performed the echo. TTE is used through the skin and the waves travel through the skin and soft tissue before reaching the heart. On the other hand, in TEE, the probe is placed directly behind the left atrium in the oesophagus and has far less tissue to penetrate. Furthermore, extreme body sizes limit the acoustic window and degrade the image quality in TTE. In addition, TTE is limited in its views, leading to better visualisation of close structures. A common example is the visibility of the left atrial appendage which is known to form clots. TTE can be utilised to test the structure and function but not the perfusion of the myocardium. This would require more advanced imaging modalities such as SPECT and PET scans, but perfusion can also be interpreted based on wall motion, which can be visualised using a TTE. Thus, TEE is useful in cases where the utilisation of TTE is limited.

In addition, this study included patients who experienced blunt chest injuries and were seen by a front-line physician in the hospital. However, many patients had blunt force chest trauma out of the hospital and died on the spot. These were not included in our study. Furthermore, the present study focussed on the initial assessment of diagnostic tests in blunt cardiac injury. The present meta-analysis did not consider follow-up measurements of cardiac biomarkers or repeated ECG recordings.

## Conclusion

This systematic review and meta-analysis support the view that the routine initial use of ECG in conjunction with troponin T offers a pragmatic and cost-effective approach to rule out blunt cardiac injury in the vast majority of cases. In addition, TTE and TEE can accurately confirm blunt cardiac injuries in suspected cases. We believe that there is a need for large prospective multicentre studies to confirm the conclusion reached in this manuscript.

*Data used for analysis are available on request from the corresponding author.

## Supplementary Information

**Additional file 1.** Supplemental Tables.

**Additional file 2.** **Supplementary Figure 1.** CPK-DOR-Forest Plot.

**Additional file 3.** **Supplementary Figure 2.** CPK-NLR-Forest Plot.

**Additional file 4.** **Supplementary Figure 3.** CPK-PLR-Forest Plot.

**Additional file 5.** **Supplementary Figure 4.** CPK-Sens-Forest Plot.

**Additional file 6.** **Supplementary Figure 5.** CPK-Spec-Forest Plot.

**Additional file 7.** **Supplementary Figure 6.** ECG-DOR-Forest Plot.

**Additional file 8.** **Supplementary Figure 7.** ECG-NLR-Forest Plot.

**Additional file 9.** **Supplementary Figure 8.** ECG-PLR-Forest Plot.

**Additional file 10.** **Supplementary Figure 9.** ECG-Sens-Forest Plot.

**Additional file 11.** **Supplementary Figure 10.** ECG-Spec-Forest Plot.

**Additional file 12.** **Supplementary Figure 11.** TropI-DOR-Forest Plot.

**Additional file 13.** **Supplementary Figure 12.** TropI-NLR-Forest Plot.

**Additional file 14.** **Supplementary Figure 13.** TropI-PLR-Forest Plot.

**Additional file 15.** **Supplementary Figure 14.** TropI-Sens-Forest Plot.

**Additional file 16.** **Supplementary Figure 15.** TropI-Spec-Forest Plot.

**Additional file 17.** **Supplementary Figure 16.** TropT-DOR-Forest Plot.

**Additional file 18.** **Supplementary Figure 17.** Trop-NLR-Forest Plot.

**Additional file 19.** **Supplementary Figure 18.** TropT-PLR-Forest Plot.

**Additional file 20.** **Supplementary Figure 19.** TropT-Sens-Forest Plot.

**Additional file 21.** **Supplementary Figure 20.** TropT-Spec-Forest Plot.

**Additional file 22.** **Supplementary Figure 21.** TTE-DOR-Forest Plot.

**Additional file 23.** **Supplementary Figure 22.** TTE-NLR-Forest Plot.

**Additional file 24.** **Supplementary Figure 23.** TTE-PLR-Forest Plot.

**Additional file 25.** **Supplementary Figure 24.** TTE-Sens-Forest Plot.

**Additional file 26.** **Supplementary Figure 25.** TTE-Spec-Forest Plot.

**Additional file 27.** **Supplementary Figure 26.** ECG Receiver Operating Characteristic (ROC) Curve Analysis.

**Additional file 28.** **Supplementary Figure 27.** Troponin I Receiver Operating Characteristic (ROC) Curve Analysis.

**Additional file 29.** **Supplementary Figure 28.** TTE Receiver Operating Characteristic (ROC) Curve Analysis.

**Additional file 30.** **Supplementary Figure 29.** Troponin T Receiver Operating Characteristic (ROC) Curve Analysis.

**Additional file 31.** **Supplementary Figure 30.** CPK-MB Receiver Operating Characteristic (ROC) Curve Analysis.

**Additional file 32.** PRISMA 2020 Checklist.

## Data Availability

Data is available on request from the corresponding author.
