# Supplementary material for: Accuracy of diagnostic tests in cardiac injury after blunt chest trauma: a systematic review and meta-analysis
Source: World J Emerg Surg. 2023 May 27;18:36. doi: 10.1186/s13017-023-00504-9 (PMC10225099; doi:10.1186/s13017-023-00504-9)
Supplement: Supplementary file 1 — Additional file 1. Supplemental Tables. [file 13017_2023_504_MOESM1_ESM.docx]

# Supplemental Tables

**Supplemental Table 1:** Search Strategy

| **#** | **Query** | **Results from 13 Oct 2022** |
| --- | --- | --- |
| 1 | exp Thoracic Injuries/ | 123,419 |
| 2 | chest injur$.ti,ab. | 4,226 |
| 3 | blunt chest trauma.ti,ab. | 4,512 |
| 4 | blunt cardiac [trauma.mp.](http://scanmail.trustwave.com/?c=261&d=053I47rh3XkxNLSyCbtUT4z3KK0cDiShpmCgecsB7A&u=http%3a%2f%2ftrauma%2emp) | 213 |
| 5 | cardiac injur$.ti,ab. | 12,763 |
| 6 | blunt chest injur$.ti,ab. | 551 |
| 7 | exp Sternum/ and exp Fractures, Bone/ | 1,812 |
| 8 | sternal fracture$.ti,ab. | 1,098 |
| 9 | 1 or 2 or 3 or 4 or 5 or 6 or 7 or 8 | 136,754 |
| 10 | exp Myocardial Contusions/ | 885 |
| 11 | myocardial contusion$.ti,ab. | 753 |
| 12 | exp Contusions/ | 26,867 |
| 13 | contusion$.ti,ab. | 26,546 |
| 14 | 10 or 11 or 12 or 13 | 41,574 |
| 15 | 9 and 14 | 6,323 |
| 16 | exp Troponin/ | 97,992 |
| 17 | Troponin.ti,ab. | 78,879 |
| 18 | 16 or 17 | 112,139 |
| 19 | exp Electrocardiography/ | 391,022 |
| 20 | ECG.ti,ab. | 185,272 |
| 21 | electrocardio$.ti,ab. | 208,042 |
| 22 | 19 or 20 or 21 | 555,607 |
| 23 | exp Radionuclide Imaging/ | 419,664 |
| 24 | Radioisotope scan$.ti,ab. | 1,157 |
| 25 | 23 or 24 | 420,040 |
| 26 | exp Echocardiography/ | 551,471 |
| 27 | echo$.ti,ab. | 638,943 |
| 28 | 26 or 27 | 835,180 |
| 29 | exp Radionuclide Angiography/ | 9,997 |
| 30 | Radionuclide angiog$.ti,ab. | 4,612 |
| 31 | 29 or 30 | 13,715 |
| 32 | exp Tomography, Emission-Computed, Single-Photon/ | 56,719 |
| 33 | Single photon emission computed tomography.ti,ab. | 33,731 |
| 34 | SPECT.ti,ab. | 79,167 |
| 35 | 32 or 33 or 34 | 106,720 |
| 36 | Multiple gated acquisition.ti,ab. | 205 |
| 37 | MUGA.ti,ab. | 981 |
| 38 | 36 or 37 | 1,088 |
| 39 | Pyrophosphate scan$.ti,ab. | 256 |
| 40 | 18 or 22 or 25 or 28 or 31 or 35 or 38 or 39 | 1,795,290 |
| 41 | 15 and 40 | 1,199 |
| 42 | limit 41 to English language | 1,010 |
| 43 | remove duplicates from 42 | 811 |

**Supplemental Table 2:** Quality Assessment of Diagnostic Accuracy Studies (QUADAS-2).

| **Number** | **Title** | **Risk of Bias** | | | | **Applicability Concerns** | | |
| --- | --- | --- | --- | --- | --- | --- | --- | --- |
|  |  | Patient Selection | Index Test | Reference Standards | Flow and timing | Patient Selection | Index Test | Reference Standards |
| 1 | Acute and long-term clinical significance of myocardial contusion following blunt thoracic trauma: results of a prospective study | Low Risk | Low Risk | Low Risk | Unclear | Low Risk | Low Risk | Low Risk |
|  |  |  |  |  |  |  |  |  |
| 2 | Evaluation of incidence, clinical significance, and prognostic value of circulating cardiac troponin I and T elevation in hemodynamically stable patients with suspected myocardial contusion after blunt chest trauma | Low Risk | Low Risk | Low Risk | Unclear | Low Risk | Low Risk | Low Risk |
|  |  |  |  |  |  |  |  |  |
| 3 | Pediatric blunt cardiac injury: epidemiology, clinical features, and diagnosis. | Unclear | Low Risk | Low Risk | Low Risk | Unclear | Low Risk | Low Risk |
|  |  |  |  |  |  |  |  |  |
| 4 | Exclusion of aortic tear in the unstable trauma patient: the utility of transesophageal echocardiography | Unclear | Low Risk | Low Risk | Unclear | Unclear | Low Risk | Low Risk |
|  |  |  |  |  |  |  |  |  |
| 5 | Attention: Cardiac contusion | Low Risk | Low Risk | Low Risk | Low Risk | Low Risk | Low Risk | Low Risk |
|  |  |  |  |  |  |  |  |  |
| 6 | Retrospective analysis of patients with sternal fracture | Low Risk | Low Risk | Low Risk | Low Risk | Low Risk | Low Risk | Low Risk |
|  |  |  |  |  |  |  |  |  |
| 7 | A retrospective study on the cardiac assessment of isolated sternal fracture patients based on radiographic and clinical outcomes | Low Risk | Low Risk | Low Risk | Unclear | Low Risk | Low Risk | Low Risk |
|  |  |  |  |  |  |  |  |  |
| 8 | Is sternum fracture a concerning clinical situation? | Unclear | Low Risk | Low Risk | Low Risk | Unclear | Low Risk | Low Risk |
|  |  |  |  |  |  |  |  |  |
| 9 | Diagnostic approach for myocardial contusion: a retrospective evaluation of patient data and review of the literature | Low Risk | Low Risk | Low Risk | Low Risk | Low Risk | Low Risk | Low Risk |
|  |  |  |  |  |  |  |  |  |
| 10 | Transesophageal Echocardiography at the Golden Hour: Identification of Blunt Traumatic Aortic Injuries in the Emergency Department | Unclear | Low Risk | Low Risk | Low Risk | Low Risk | Low Risk | Low Risk |
|  |  |  |  |  |  |  |  |  |
| 11 | Cardiac findings of sternal fractures due to thoracic trauma: A five-year retrospective study | Unclear | Low Risk | Low Risk | Low Risk | Unclear | Low Risk | Low Risk |
|  |  |  |  |  |  |  |  |  |
| 12 | The Feasibility of Dual-Energy Computed Tomography in Cardiac Contusion Imaging for Mildest Blunt Cardiac Injury | Low Risk | Low Risk | Low Risk | Low Risk | Low Risk | Low Risk | Low Risk |
|  |  |  |  |  |  |  |  |  |
| 13 | The Role of Troponin in Blunt Cardiac Injury After Multiple Trauma in Humans | Unclear | Low Risk | Low Risk | Low Risk | Unclear | Low Risk | Low Risk |
|  |  |  |  |  |  |  |  |  |
| 14 | Management of pericardial fluid in blunt trauma: variability in practice and predictors of operative outcome in patients with computed tomography evidence of pericardial fluid | Unclear | Low Risk | Low Risk | Low Risk | Unclear | Low Risk | Low Risk |
|  |  |  |  |  |  |  |  |  |
| 15 | Evaluation of Myocardial Injury using Standard Diagnostic Tools and Tissue Doppler Imaging in Blunt Trauma Chest | Low Risk | Low Risk | Low Risk | Low Risk | Low Risk | Low Risk | Low Risk |
|  |  |  |  |  |  |  |  |  |
| 16 | Imaging in blunt cardiac injury: Computed tomographic findings in cardiac contusion and associated injuries | Unclear | Low Risk | Low Risk | Low Risk | Unclear | Low Risk | Low Risk |
|  |  |  |  |  |  |  |  |  |
| 17 | Troponin T in Patients with Traumatic Chest Injuries with and without Cardiac Involvement: Insights from an Observational Study | Low Risk | Low Risk | Low Risk | Low Risk | Low Risk | Low Risk | Low Risk |
|  |  |  |  |  |  |  |  |  |
|  |  |  |  |  |  |  |  |  |
| 18 | Estimated Incidence of Cardiac Contusion Using Transthoracic Echocardiography in Patients Suffering from Severe Blunt Trauma to the Chest | Low Risk | Low Risk | Low Risk | Low Risk | Low Risk | Low Risk | Low Risk |
|  |  |  |  |  |  |  |  |  |
| 19 | Investigation of myocardial contusion with sternal fracture in the emergency department | Low Risk | Low Risk | Low Risk | Unclear | Low Risk | Low Risk | Low Risk |
|  |  |  |  |  |  |  |  |  |
| 20 | Location of Sternal Fractures as a Possible Marker for Associated Injuries | Low Risk | Low Risk | Low Risk | Low Risk | Low Risk | Low Risk | Low Risk |
|  |  |  |  |  |  |  |  |  |
| 21 | Blunt traumatic cardiac rupture: therapeutic options and outcomes | Unclear | Low Risk | Low Risk | Low Risk | Unclear | Low Risk | Low Risk |
|  |  |  |  |  |  |  |  |  |
| 22 | Blunt rupture of the heart: surgical treatment of three different clinical presentations. | Unclear | Low Risk | Low Risk | Low Risk | Unclear | Low Risk | Low Risk |
|  |  |  |  |  |  |  |  |  |
| 23 | Heterogeneity of traumatic injury of the tricuspid valve: A report of four cases | Unclear | Low Risk | Low Risk | Low Risk | Unclear | Low Risk | Low Risk |
|  |  |  |  |  |  |  |  |  |
| 24 | The relevance of the detection of troponins to the forensic diagnosis of cardiac contusion | Low Risk | Low Risk | Low Risk | Low Risk | Low Risk | Low Risk | Low Risk |
|  |  |  |  |  |  |  |  |  |
| 25 | Sternal Fracture Should Prompt the Evaluation of the Entire Spine in Trauma Patients | Low Risk | Low Risk | Low Risk | Low Risk | Low Risk | Low Risk | Low Risk |
|  |  |  |  |  |  |  |  |  |
| 26 | Incidence and significance of cardiac troponin I release in severe trauma patients | Low Risk | Low Risk | Low Risk | Low Risk | Low Risk | Low Risk | Low Risk |
|  |  |  |  |  |  |  |  |  |
| 27 | Cardiac troponin I as a predictor of arrhythmia and ventricular dysfunction in trauma patients with myocardial contusion | Low Risk | Low Risk | Low Risk | Low Risk | Low Risk | Low Risk | Low Risk |
|  |  |  |  |  |  |  |  |  |
| 28 | The usefulness of serum troponin levels in evaluating cardiac injury | Unclear | Low Risk | Low Risk | Low Risk | Unclear | Low Risk | Low Risk |
|  |  |  |  |  |  |  |  |  |
| 29 | Creatinine-kinase-MB determination in non-cardiac trauma: its difference with cardiac infarction and its restricted use in trauma situations | Low Risk | Low Risk | Low Risk | Low Risk | Low Risk | Low Risk | Low Risk |
|  |  |  |  |  |  |  |  |  |
| 30 | Sternal fractures: retrospective analysis of 100 cases | Low Risk | Low Risk | Low Risk | Low Risk | Low Risk | Low Risk | Low Risk |
|  |  |  |  |  |  |  |  |  |
| 31 | Acute and Long-Term Clinical Significance of Myocardial Contusion following Blunt Thoracic Trauma: Results of a Prospective Study | Low Risk | Low Risk | Low Risk | Low Risk | Low Risk | Low Risk | Low Risk |
|  |  |  |  |  |  |  |  |  |
| 32 | Echocardiogram in sternal fracture | Low Risk | Low Risk | Low Risk | Low Risk | Low Risk | Low Risk | Low Risk |
|  |  |  |  |  |  |  |  |  |
| 33 | Cardiac contusion in blunt chest trauma: a combined study of transesophageal echocardiography and cardiac troponin I determination | Low Risk | Low Risk | Low Risk | Low Risk | Low Risk | Low Risk | Low Risk |
|  |  |  |  |  |  |  |  |  |
| 34 | The Incidence of Myocardial Contusion in 160 Patients with Blunt Chest Trauma Diagnostic Criteria and Outcome | Low Risk | Low Risk | Low Risk | Low Risk | Low Risk | Low Risk | Low Risk |
|  |  |  |  |  |  |  |  |  |
| 35 | Myocardial contusion as a result of isolated sternal fractures: a fact or a myth? | Low Risk | Low Risk | Low Risk | Low Risk | Low Risk | Low Risk | Low Risk |
|  |  |  |  |  |  |  |  |  |
| 36 | Coronary dissection and myocardial infarction following blunt chest trauma | Unclear | Low Risk | Low Risk | Low Risk | Unclear | Low Risk | Low Risk |
|  |  |  |  |  |  |  |  |  |
| 37 | Circulating cardiac troponin I in trauma patients without cardiac contusion | Unclear | Low Risk | Low Risk | Low Risk | Unclear | Low Risk | Low Risk |
|  |  |  |  |  |  |  |  |  |
| 38 | Troponin I, troponin T, CKMB-activity and CKMB-mass as markers for the detection of myocardial contusion in patients who experienced blunt trauma | Low Risk | Low Risk | Low Risk | Low Risk | Low Risk | Low Risk | Low Risk |
|  |  |  |  |  |  |  |  |  |
| 39 | Early detection of myocardial contusion in patients with blunt chest trauma | Low Risk | Low Risk | Low Risk | Low Risk | Low Risk | Low Risk | Low Risk |
|  |  |  |  |  |  |  |  |  |
| 40 | Blunt cardiac injury: a 10 year institutional review | Low Risk | Low Risk | Low Risk | Low Risk | Low Risk | Low Risk | Low Risk |
|  |  |  |  |  |  |  |  |  |
| 41 | Cardiac troponin I in pediatrics: normal values and potential use in the assessment of cardiac injury | Low Risk | Low Risk | Low Risk | Low Risk | Low Risk | Low Risk | Low Risk |
|  |  |  |  |  |  |  |  |  |
| 42 | Recognition and management of nonpenetrating cardiac trauma in children | Unclear | Low Risk | Low Risk | Low Risk | Unclear | Low Risk | Low Risk |
|  |  |  |  |  |  |  |  |  |
| 43 | Improved detection of cardiac contusion with cardiac troponin I | Low Risk | Low Risk | Low Risk | Low Risk | Low Risk | Low Risk | Low Risk |
|  |  |  |  |  |  |  |  |  |
| 44 | Usefulness of transthoracic and transoesophageal echocardiography in recognition and management of cardiovascular injuries after blunt chest trauma | Low Risk | Low Risk | Low Risk | Low Risk | Low Risk | Low Risk | Low Risk |
|  |  |  |  |  |  |  |  |  |
| 45 | The usefulness of transesophageal echocardiography in diagnosing cardiac contusions | Unclear | Low Risk | Low Risk | Low Risk | Unclear | Low Risk | Low Risk |
|  |  |  |  |  |  |  |  |  |
| 46 | Systematic transesophageal echocardiography for detection of mediastinal lesions in patients with multiple injuries | Low Risk | Low Risk | Low Risk | Low Risk | Low Risk | Low Risk | Low Risk |
|  |  |  |  |  |  |  |  |  |
| 47 | Limiting cardiac evaluation in patients with suspected myocardial contusion | Low Risk | Low Risk | Low Risk | Unclear | Low Risk | Low Risk | Low Risk |
|  |  |  |  |  |  |  |  |  |
| 48 | Isolated sternal fracture: an audit of 10 years' experience | Low Risk | Low Risk | Low Risk | Low Risk | Low Risk | Low Risk | Low Risk |
|  |  |  |  |  |  |  |  |  |
| 49 | The role of echocardiography in blunt chest trauma: a transthoracic and transesophageal echocardiographic study | Unclear | Low Risk | Low Risk | Low Risk | Unclear | Low Risk | Low Risk |
|  |  |  |  |  |  |  |  |  |
| 50 | Cardiac involvement in seatbelt-related and direct sternal trauma: a prospective study and management implications | Low Risk | Low Risk | Low Risk | Unclear | Low Risk | Low Risk | Low Risk |
|  |  |  |  |  |  |  |  |  |
| 51 | Diagnosis of myocardial contusion | Low Risk | Low Risk | Low Risk | Low Risk | Low Risk | Low Risk | Low Risk |
|  |  |  |  |  |  |  |  |  |

**Supplemental Table 3:** Study characteristics of included studies

| **Rank** | **Title** | **First Author** | **Year** | **Journal** | **Number of patients** | **Cardiac injuries** |
| --- | --- | --- | --- | --- | --- | --- |
| 1 | Acute and long-term clinical significance of myocardial contusion following blunt thoracic trauma: results of a prospective study | Michael Lindstaedt | 2002 | Journal of Trauma, Injury, Infection, and Critical Care | 118 | 14 |
| 2 | Evaluation of incidence, clinical significance, and prognostic value of circulating cardiac troponin I and T elevation in hemodynamically stable patients with suspected myocardial contusion after blunt chest trauma | J P Bertinchant | 2000 | J Journal of Trauma, Injury, Infection, and Critical Care | 94 | 26 |
| 3 | Pediatric blunt cardiac injury: epidemiology, clinical features, and diagnosis. | M D Dowd | 1996 | Journal of Trauma, Injury, Infection, and Critical Care | 184 | 184 |
| 4 | Exclusion of aortic tear in the unstable trauma patient: the utility of transesophageal echocardiography | S M Cohn | 1995 | J Journal of Trauma, Injury, Infection, and Critical Care | 53 | 1 |
| 5 | Attention: Cardiac contusion | E S Guermen | 2022 | Ulus Travma Acil Cerrahi Derg | 65 | 29 |
| 6 | Retrospective analysis of patients with sternal fracture | Sule Yakar | 2021 | Turkish Journal of Emergency Medicine | 128 | 12 |
| 7 | A retrospective study on the cardiac assessment of isolated sternal fracture patients based on radiographic and clinical outcomes | M Ahmadinejad | 2021 | Annals of Medicine and Surgery | 61 | 9 |
| 8 | Is sternum fracture a concerning clinical situation? | O Guler | 2021 | Annals of Clinical and Analytical Medicine | 56 | 1 |
| 9 | Diagnostic approach for myocardial contusion: a retrospective evaluation of patient data and review of the literature | E MMV Lieshout | 2021 | European Journal of Trauma and Emergency Surgery | 117 | 35 |
| 10 | Transoesophageal echocardiography at the golden hour: identification of blunt traumatic aortic injuries in the emergency department | A Osman | 2020 | The Journal of Emergency Medicine | 5 | 5 |
| 11 | Cardiac findings of sternal fractures due to thoracic trauma: A five-year retrospective study | A Ulusan | 2018 | Turkish Journal of Trauma & Emergency Surgery | 72 | 4 |
| 12 | The Feasibility of dual-energy computed tomography in cardiac contusion imaging for mildest blunt cardiac injury | R Sade | 2017 | Journal of Computer Assisted Tomography | 17 | 14 |
| 13 | The roae of troponin in blunt cardiac injury ahfter multiple trauma in Humans | M Kalbitz | 2017 | World Journal of Surgery | 173 | 51 |
| 14 | Management of pericardial fluid in blunt trauma: variability in practice and predictors of operative outcome in patients with computed tomography evidence of pericardial fluid | CE Witt | 2018 | Journal of Trauma, Injury, Infection, and Critical Care | 75 | 6 |
| 15 | Evaluation of myocardial injury using standard diagnostic tools and tissue Doppler imaging in blunt trauma chest | PL Gautam | 2017 | Journal of cCinical and Diagnostic Research | 30 | 11 |
| 16 | Imaging in blunt cardiac injury: Computed tomographic findings in cardiac contusion and associated injuries | MH Hammer | 2016 | Injury | 42 | 42 |
| 17 | Troponin T in patients with traumatic chest injuries with and without cardiac involvement: insights from an observational study | I Mahmood | 2016 | North American Journal of Medical sciences | 993 | 20 |
| 18 | Estimated incidence of cardiac contusion using pransthoracic echocardiography in Patients suffering from severe blunt trauma to the chest | A Bahar | 2014 | Acta Chirurgica Belgica | 210 | 4 |
| 19 | Investigation of myocardial contusion with sternal fracture in the emergency department | J Audette | 2014 | Can Fam Physician | 54 | 2 |
| 20 | Location of sternal fractures as a possible marker for associated injuries | M Scheyerer | 2013 | Emergency Medicine International | 58 | 5 |
| 21 | Blunt traumatic cardiac rupture: therapeutic options and outcomes | Yu-Yun nan | 2009 | Injury | 11 | 11 |
| 22 | Blunt rupture of the heart: surgical treatment of three different clinical presentations. | A Seguin | 2008 | Journal of Trauma, Injury, Infection, and Critical Care | 3 | 3 |
| 23 | Heterogeneity of traumatic injury of the tricuspid valve: A report of four cases | I Schuster | 2008 | Wiener Klinische Wochenschrift | 4 | 4 |
| 24 | The relevance of the detection of troponins to the forensic diagnosis of cardiac contusion | J Peter | 2006 | Forensic Science ISternational Supplement series | 36 | 25 |
| 25 | Sternal fracture should prompt the evaluation of the entire spine in trauma patients | B Kessel | 2005 | European Journal of Trauma | 19 | 3 |
| 26 | Incidence and significance of cardiac troponin I release in severe trauma patients | A R Edouard | 2004 | Anesthesiology | 728 | 35 |
| 27 | Cardiac troponin I as a predictor of arrhythmia and ventricular dysfunction in trauma patients with myocardial contusion | G P Rajan | 2004 | Journal of Trauma, Injury, Infection, and Critical Care | 187 | 63 |
| 28 | The usefulness of serum troponin levels in evaluating cardiac injury | J N Collins | 2001 | The American Surgeon. | 66 | 2 |
| 29 | Creatinine-kinase-MB determination in non-cardiac trauma: its difference with cardiac infarction and its restricted use in trauma situations | M Ruppert | 2001 | European Journal of Emergency Medicine | 25 | 0 |
| 30 | Sternal fractures: retrospective analysis of 100 cases | K Athanassiadi | 2002 | World Journal of Surgery | 100 | 4 |
| 31 | Acute and long-term clinical significance of myocardial contusion following blunt thoracic trauma: results of apProspective study | M Lindstaedt | 2002 | Journal of Trauma, Injury, Infection, and Critical Care | 118 | 14 |
| 32 | Echocardiogram in sternal fracture | Y Wiener | 2001 | The American Journal of Emergency Medicine | 50 | 3 |
| 33 | Cardiac contusion in blunt chest trauma: a combined study of transesophageal echocardiography and cardiac troponin I determination | F Mori | 2001 | Italian Heart Journal : Official Journal of the Italian Federation of Cardiology. | 32 | 6 |
| 34 | The incidence of myocardial contusion in 160 patients with blunt chest trauma diagnostic criteria and outcome | U Boeke | 2000 | European Journal of Trauma | 160 | 27 |
| 35 | Myocardial contusion as a result of isolated sternal fractures: a fact or a myth? | E N Yilmaz | 1999 | European Journal of Emergency Medicine | 86 | 0 |
| 36 | Coronary dissection and myocardial infarction following blunt chest trauma | M Fu | 1999 | Journal of the Formosan Medical Association | 3 | 3 |
| 37 | Circulating cardiac troponin I in trauma patients without cardiac contusion | A R Edouard | 1998 | Intensive Care Medicine | 17 | 0 |
| 38 | Troponin I, troponin T, CKMB-activity and CKMB-mass as markers for the detection of myocardial contusion in patients who experienced blunt trauma | J C Swaanenburg | 1998 | International Journal of Clinical Chemistry | 89 | 3 |
| 39 | Early detection of myocardial contusion in patients with blunt chest trauma | M Lin | 1997 | Acta Cardiologica Sinica | 98 | 32 |
| 40 | Blunt cardiac injury: a 10 year institutional review | M.H Van Wijngaarden | 1997 | Injury | 70 | 70 |
| 41 | Cardiac troponin I in paediatrics: normal values and potential use in the assessment of cardiac injury | R Hirsch | 1997 | The Journal of pediatrics | 7 | 4 |
| 42 | Recognition and management of nonpenetrating cardiac trauma in children | B I Bromberg | 1996 | The Journal of pediatrics | 8 | 8 |
| 43 | Improved detection of cardiac contusion with cardiac troponin I | J E Adams | 1996 | American Heart Journal | 44 | 6 |
| 44 | Usefulness of transthoracic and transoesophageal echocardiography in recognition and management of cardiovascular injuries after blunt chest trauma | F Chirillo | 1996 | Heart | 134 | 45 |
| 45 | The usefulness of transoesophageal echocardiography in diagnosing cardiac contusions | R L Weiss | 1996 | Chest | 22 | 22 |
| 46 | Systematic transoesophageal echocardiography for detection of mediastinal lesions in patients with multiple injuries | P Catoire | 1995 | Journal of Trauma, Injury, Infection, and Critical Care | 70 | 13 |
| 47 | Limiting cardiac evaluation in patients with suspected myocardial contusion | J J Fildes | 1995 | The American Surgeon | 93 | 14 |
| 48 | Isolated sternal fracture: an audit of 10 years' experience | G J Peek | 1995 | Injury | 162 | 3 |
| 49 | The role of echocardiography in blunt chest trauma: a transthoracic and transoesophageal echocardiographic study | D G Karalis | 1994 | Journal of Trauma, Injury, Infection, and Critical Care | 105 | 31 |
| 50 | Cardiac involvement in seatbelt-related and direct sternal trauma: a prospective study and management implications | F A BuLock | 1994 | European Heart Journal | 60 | 42 |
| 51 | Diagnosis of myocardial contusion | R F Paone | 1993 | Southern Medical jJurnal | 147 | 11 |

**Supplemental Table 4:** Detailed information on the diagnostic accuracy of ECG

| ECG | |
| --- | --- |
| 28 Studies included in the meta-analysis | |
| ECG_DOR_ Forrestplot  DOR = Diagnostics Odds Ratio  DOR 5.975 (95%CI: 2.675-13.349)  I2 (=Heterogeneity) = 59.35%, P<0.01 | ECG_NLR_ Forrestplot  NLR = Negative Likelyhood Ratio  0.494 (95%CI: 0.309-0.789)  I2 (=Heterogeneity) = 64.93%, P<0.001 |
| ECG_PLR_ Forrestplot  PLR = Positive Likelyhood Ratio 2.719 (95%CI: 1.654-4.469)  I2 (=Heterogeneity) = 69.13%, P<0.001 | ECG_SENS_ Forrestplot  Sens = Sensitivity  55.1% (95%CI: 45.2-64.6)  I2 (=Heterogeneity) = 68.60%, P<0.001 |
| ECG_SPEC_ Forrestplot  Spec = Specificity  84.5% (95%CI: 74.6-91.0)  I2 (=Heterogeneity) = 68.72%, P<0.001 | |

**Supplemental Table 5:** Detailed information on the diagnostic accuracy of Troponin I

| Troponin I | |
| --- | --- |
| 17 Studies included in the Meta-analysis | |
| TropI_DOR_ Forrestplot  DOR = Diagnostics Odds Ratio  DOR 15.009 (95%CI: 6.910 – 32.603)  I2 (=Heterogeneity) = 3332%, P=0.090 | TropI _NLR_ Forrestplot  NLR = Negative Likelyhood Ratio  0.329 (95%CI: 0.170-0.638)  I2 (=Heterogeneity) = 8962%, P<0.01 |
| TropI _PLR_ Forrestplot  PLR = Positive Likelyhood Ratio 3.792 (95%CI: 2.181 – 6.594)  I2 (=Heterogeneity) = 8435%, P<0.001 | TropI _SENS_ Forrestplot  Sens = Sensitivity  64.4% (95%CI: 52.3-74.9)  I2 (=Heterogeneity) = 4534%, P=0.022 |
| TropI _SPEC_ Forrestplot  Spec = Specificity  84.1% (95%CI: 72.3-91.5)  I2 (=Heterogeneity) = 8579%, P<0.001 | |

**Supplemental Table 6:** Detailed information on the diagnostic accuracy of Troponin T

| Troponin T | |
| --- | --- |
| 6 Studies included in the Meta-analysis | |
| TropT_DOR_ Forrestplot  DOR = Diagnostics Odds Ratio  DOR 16.048 (95%CI: 7.453 - 34.558)  I2 (=Heterogeneity) = 2454%, P=0.250 | TropT _NLR_ Forrestplot  NLR = Negative Likelyhood Ratio  0.217 (95%CI: 0.120-0.395)  I2 (=Heterogeneity) = 7564%, P<0.001 |
| TropT _PLR_ Forrestplot  PLR = Positive Likelyhood Ratio 4.368 (95%CI: 2.473 – 7.715)  I2 (=Heterogeneity) = 7315%, P=0.002 | TropT _SENS_ Forrestplot  Sens = Sensitivity  68.4% (95%CI: 40.2-87.5)  I2 (=Heterogeneity) = 8440%, P<0.001 |
| TropT _SPEC_ Forrestplot  Spec = Specificity  85.8% (95%CI: 73.6-92.9)  I2 (=Heterogeneity) = 8394%, P<0.001 | |

**Supplemental Table 7:** Detailed information on the diagnostic accuracy of CK-MB

| CK-MB | |
| --- | --- |
| 22 Studies included in the Meta-analysis | |
| CPK_DOR_ Forrestplot  DOR = Diagnostics Odds Ratio  DOR 3.598 (95%CI: 1.832 – 7.068)  I2 (=Heterogeneity) = 5048%, P=0.004 | CPK _NLR_ Forrestplot  NLR = Negative Likelyhood Ratio  0.523 (95%CI: 0.386-0.710)  I2 (=Heterogeneity) = 5176%, P=0.003 |
| CPK _PLR_ Forrestplot  PLR = Positive Likelyhood Ratio 1.927 (95%CI: 1.370 – 2.711)  I2 (=Heterogeneity) = 6163%, P<0.001 | CPK _SENS_ Forrestplot  Sens = Sensitivity  55.2% (95%CI: 43.1-66.6)  I2 (=Heterogeneity) = 6555%, P<0.001 |
| CPK _SPEC_ Forrestplot  Spec = Specificity  75.8% (95%CI: 62.7-85.4)  I2 (=Heterogeneity) = 8754%, P<0.001 | |

**Supplemental Table 8:** Detailed information on the diagnostic accuracy of TransThoracic Echocardiography (TTE)

| TransThoracic Echocardiography (TTE) | |
| --- | --- |
| 20 Studies included in the Meta-analysis | |
| TTE_DOR_ Forrestplot  DOR = Diagnostics Odds Ratio  DOR 10.077 (95%CI: 3.845 – 26.408)  I2 (=Heterogeneity) = 3888%, P=0.040 | TTE _NLR_ Forrestplot  NLR = Negative Likelyhood Ratio  0.446 (95%CI: 0.249-0.799)  I2 (=Heterogeneity) = 4777%, P=0.009 |
| TTE _PLR_ Forrestplot  PLR = Positive Likelyhood Ratio 3.558 (95%CI: 1.967 – 6.436)  I2 (=Heterogeneity) = 4941%, P=0.007 | TTE _SENS_ Forrestplot  Sens = Sensitivity  47.0% (95%CI: 34.2-60.2)  I2 (=Heterogeneity) = 7464%, P<0.001 |
| TTE _SPEC_ Forrestplot  Spec = Specificity  91.4% (95%CI: 84.3-95.5)  I2 (=Heterogeneity) = 4857%, P=0.008 | |

**Supplemental Table 9a:** Detailed information on the diagnostic accuracy of Transoesophageal Echocardiography (TEE) including case reports

| Transoesophageal Echocardiography (TEE) | |
| --- | --- |
| 5 Studies included in the Meta-analysis (including case reports) | |
| TEE_DOR_ Forrestplot DOR = Diagnostics Odds Ratio DOR 59.994 (95%CI: 1.947 – 1848.814) I2 (=Heterogeneity) = 69.24%, P=0.011 | TEE _NLR_ Forrestplot NLR = Negative Likelihood Ratio 0.131 (95%CI: 0.034-0.513) I2 (=Heterogeneity) = 51.43%, P=0.083 |
| TEE _PLR_ Forrestplot PLR = Positive Likelihood Ratio 5.711 (95%CI: 1.065 – 30.613) I2 (=Heterogeneity) = 67.97%, P<0.014 | TEE _SENS_ Forrestplot Sens = Sensitivity 93.1% (95%CI: 76.5-98.2) I2 (=Heterogeneity) = 24.63%, P=0.257 |
| TTE _SPEC_ Forrestplot Spec = Specificity 87.6% (95%CI: 46.1-98.3) I2 (=Heterogeneity) = 54.83%, P=0.065 | |

**Supplemental Table 9b:** Detailed information on the diagnostic accuracy of Transoesophageal Echocardiography (TTE) excluding case reports

| Transoesophageal Echocardiography (TEE) |
| --- |
| 3 Studies included in the analysis (excluding case reports) |
| Sensitivity: 0.867 (range 0.400 – 0.992)  Specificity: 0.721 (range 0.358 – 0.982) |

**Supplemental Table 10:** Journals in which the articles were published

| **Journal** | **Journal quartile** | **Journal country** | **Number of articles** |
| --- | --- | --- | --- |
| Journal of Trauma, Injury, infection, and Critical Care | Q2 | USA | 10 |
| Injury | Q3 | Netherlands | 4 |
| European Journal of Emergency Medicine | Q1 | UK | 2 |
| European Journal of Trauma | Q1 | Germany | 2 |
| The American Surgeon | Q4 | USA | 2 |
| The Journal of Pediatrics | Q1 | USA | 2 |
| World Journal of Surgery | Q2 | USA | 2 |
| Intensive Care Medicine | Q1 | USA | 1 |
| The American Journal of Emergency Medicine | Q1 | USA | 1 |
| Annals of Medicine and Surgery | Q2 | UK | 1 |
| Acta Cardiologica Sinica | Q3 | Taiwan | 1 |
| Acta Chirurgica Belgica | Q4 | UK | 1 |
| American Heart Journal | Q1 | USA | 1 |
| Anesthesiology | Q1 | USA | 1 |
| Annals of Clinical and Analytical Medicine | Q4 | Turkey | 1 |
| Can Fam Physician | Q2 | Canada | 1 |
| Chest | Q1 | USA | 1 |
| Emergency Medicine International | Q2 | Egypt | 1 |
| European Heart Journal | Q1 | UK | 1 |
| European Journal of Trauma and Emergency Surgery | Q3 | Germany | 1 |
| Forensic Science International Supplement Series | Q2 | Ireland | 1 |
| Heart | Q1 | UK | 1 |
| International Journal of Clinical Chemistry | Q1 | Netherlands | 1 |
| Italian Heart Journal: Official Journal of the Italian Federation of Cardiology. | Q4 | Italy | 1 |
| Journal of Clinical and Diagnostic Research | Q4 | India | 1 |
| Journal of Computer Assisted Tomography | Q3 | Turkey | 1 |
| Journal of the Formosan Medical Association | Q1 | Singapore | 1 |
| North American Journal of Medical Sciences | Q2 | India | 1 |
| Southern Medical Journal | Q4 | USA | 1 |
| The Journal of Emergency Medicine | Q4 | USA | 1 |
| Turkish Journal of Emergency Medicine | Q1 | Turkey | 1 |
| Turkish Journal of Trauma & Emergency Surgery | Q4 | Turkey | 1 |
| Ulus Travma Acil Cerrahi Derg | Q3 | Turkey | 1 |
| Wiener Klinische Wochenschrift | Q3 | Austria | 1 |

**Supplemental Table 11:** Authors countries of origin

| **First author** | **Author country** |
| --- | --- |
| I Schuster | Austria |
| M Ruppert | Belgium |
| J Audette | Canada |
| A R Edouard | France |
| A R Edouard | France |
| A Seguin | France |
| J P Bertinchant | france |
| P Catoire | France |
| J Peter | Germany |
| M Kalbitz | Germany |
| M Lindstaedt | Germany |
| M Lindstaedt | Germany |
| U Boeke | Germany |
| K Athanassiadi | Greece |
| PL Gautam | India |
| A Bahar | Iran |
| M Ahmadinejad | Iran |
| B Kessel | Israel |
| Y Wiener | Israel |
| F Chirillo | Italy |
| F Mori | Italy |
| A Osman | Malaysia |
| E MMV Lieshout | Netherlands |
| E N Yilmaz | Netherlands |
| J C Swaanenburg | Netherlands |
| I Mahmood | Qatar |
| G P Rajan | Switzerlad |
| M Scheyerer | Switzerlad |
| M Fu | Taiwan |
| M Lin | Taiwan |
| Yu-Yun nan | Taiwan |
| A Ulusan | Turkey |
| E S Guermen | Turkey |
| O Guler | Turkey |
| R Sade | Turkey |
| S Yakar | Turkey |
| F A BuLock | UK |
| G J Peek | UK |
| B I Bromberg | USA |
| CE Witt | USA |
| D G Karalis | USA |
| J E Adams | USA |
| J J Fildes | USA |
| J N Collins | USA |
| M D Dowd | USA |
| M.H Van Wijngaarden | USA |
| MH Hammer | USA |
| R F Paone | USA |
| R Hirsch | USA |
| R L Weiss | USA |
| S M Cohn | USA |
