# Supplementary figures and images for: Accuracy of diagnostic tests in cardiac injury after blunt chest trauma: a systematic review and meta-analysis
Source: World J Emerg Surg. 2023 May 27;18:36. doi: 10.1186/s13017-023-00504-9 (PMC10225099; doi:10.1186/s13017-023-00504-9)

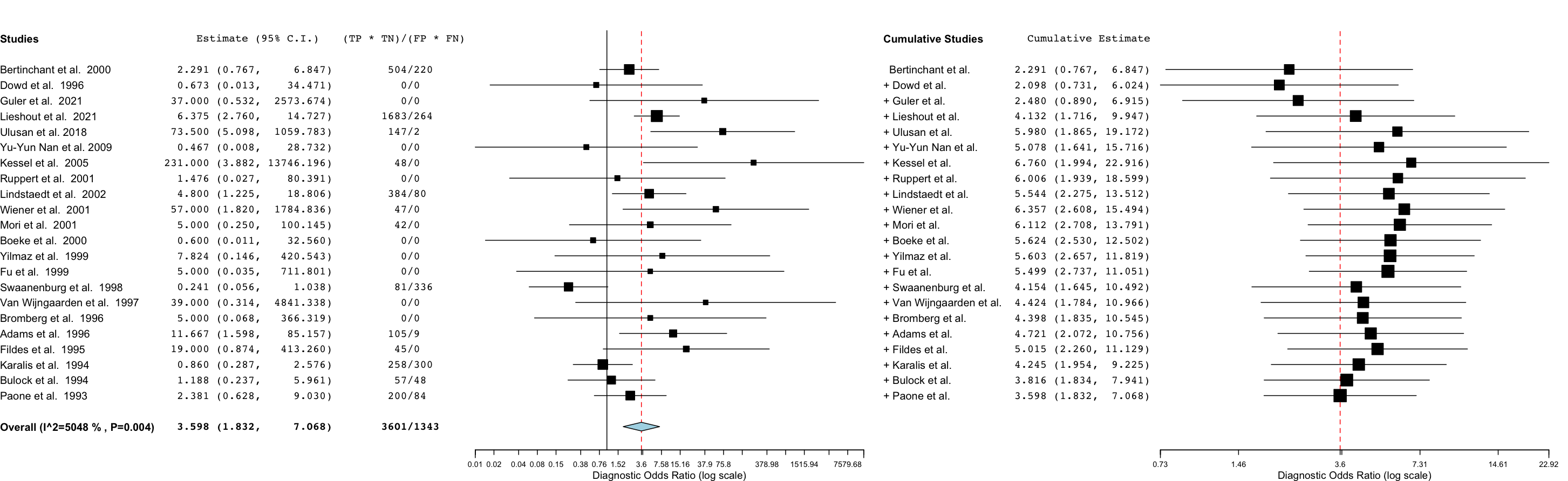

Supplement: Supplementary file 2 — Additional file 2. Supplementary Figure 1. CPK-DOR-Forest Plot. [file 13017_2023_504_MOESM2_ESM.png]

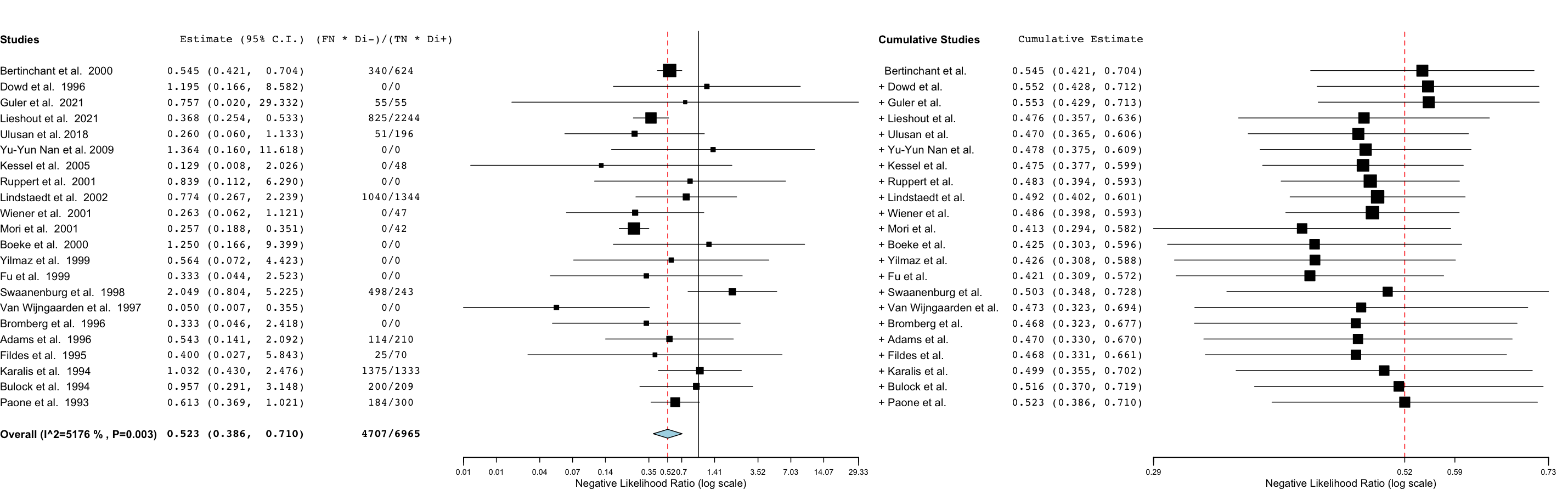

Supplement: Supplementary file 3 — Additional file 3. Supplementary Figure 2. CPK-NLR-Forest Plot. [file 13017_2023_504_MOESM3_ESM.png]

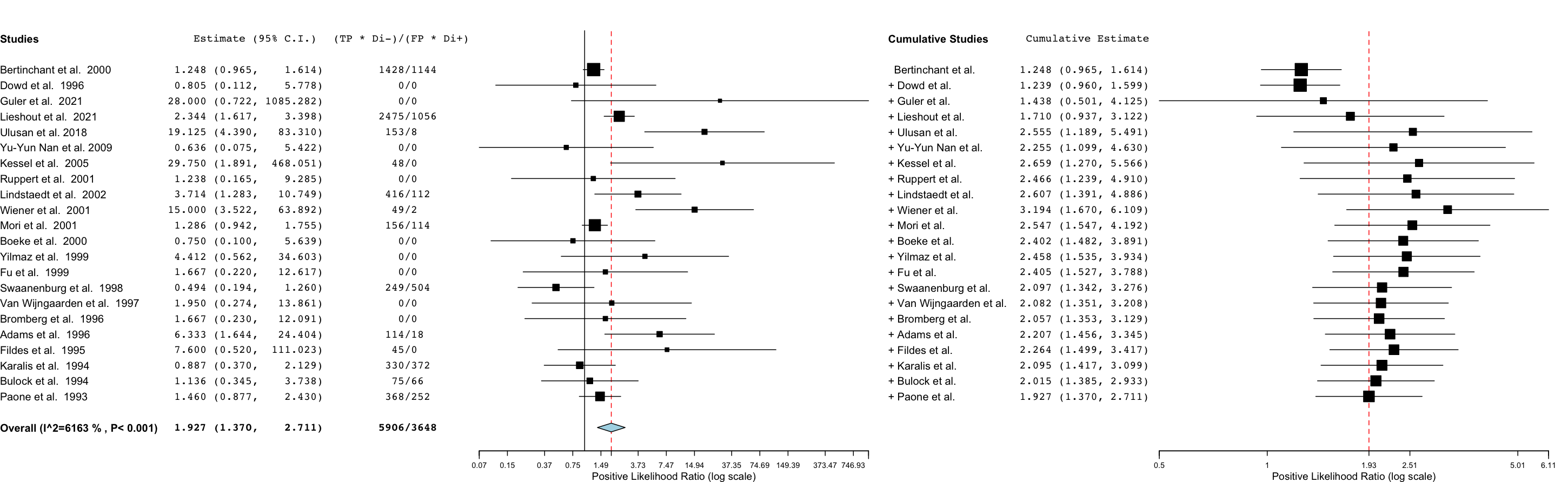

Supplement: Supplementary file 4 — Additional file 4. Supplementary Figure 3. CPK-PLR-Forest Plot. [file 13017_2023_504_MOESM4_ESM.png]

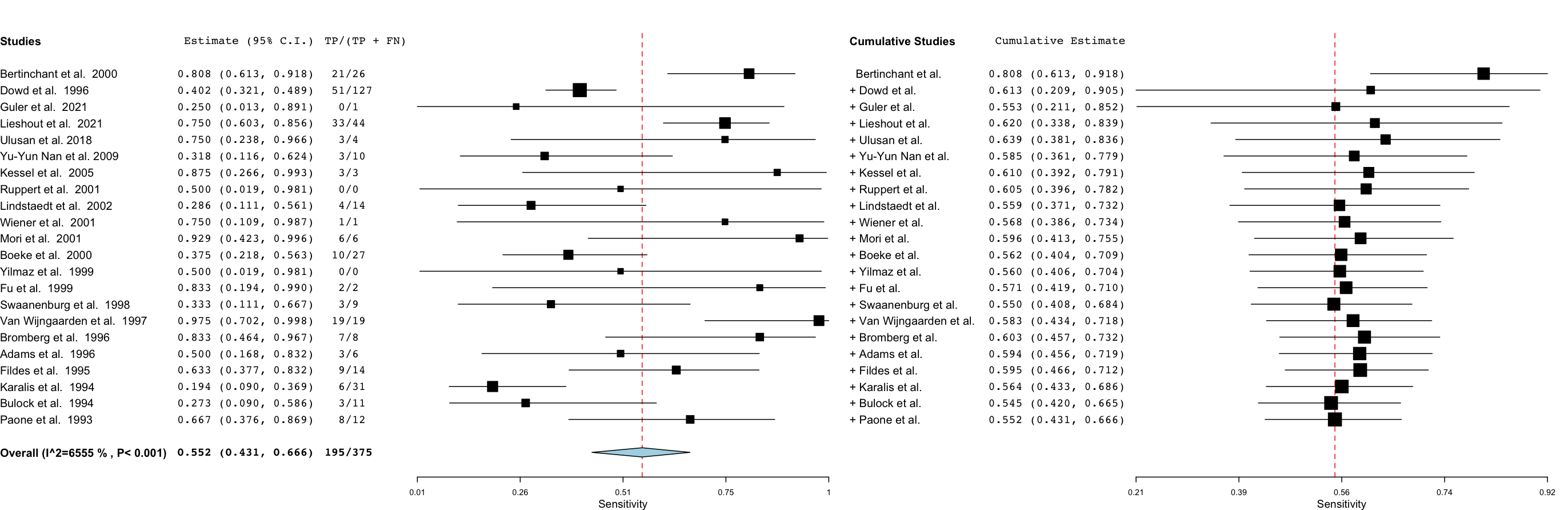

Supplement: Supplementary file 5 — Additional file 5. Supplementary Figure 4. CPK-Sens-Forest Plot. [file 13017_2023_504_MOESM5_ESM.png]

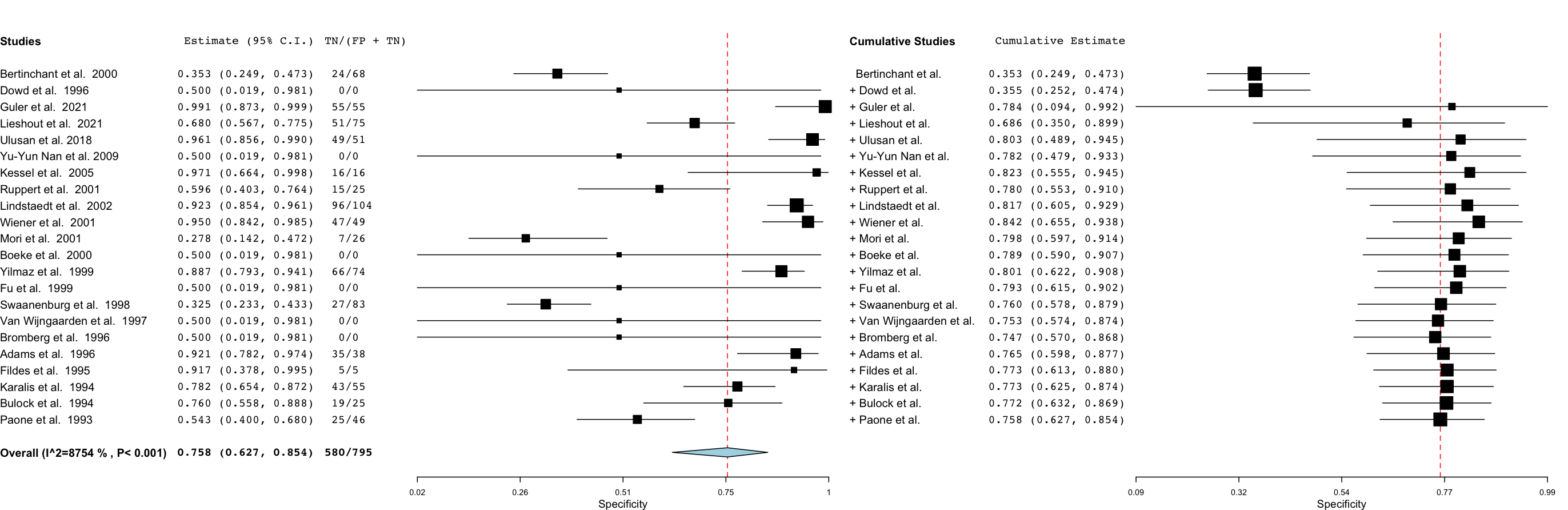

Supplement: Supplementary file 6 — Additional file 6. Supplementary Figure 5. CPK-Spec-Forest Plot. [file 13017_2023_504_MOESM6_ESM.png]

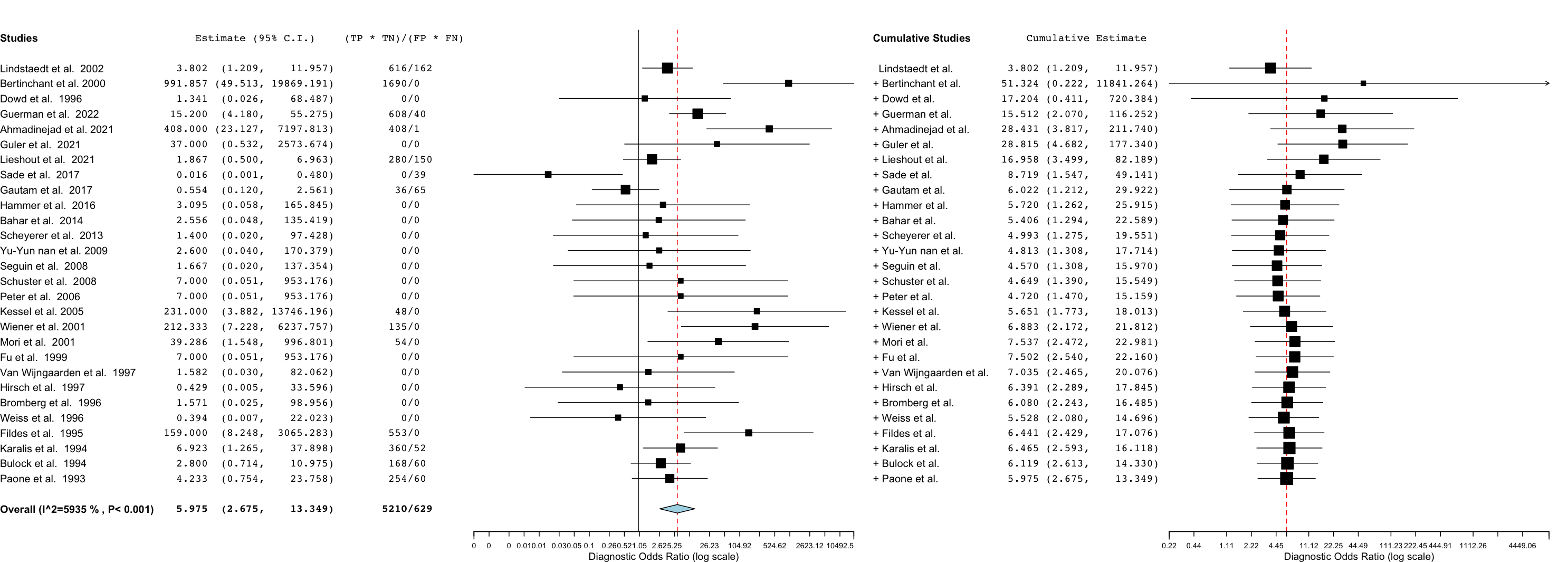

Supplement: Supplementary file 7 — Additional file 7. Supplementary Figure 6. ECG-DOR-Forest Plot. [file 13017_2023_504_MOESM7_ESM.png]

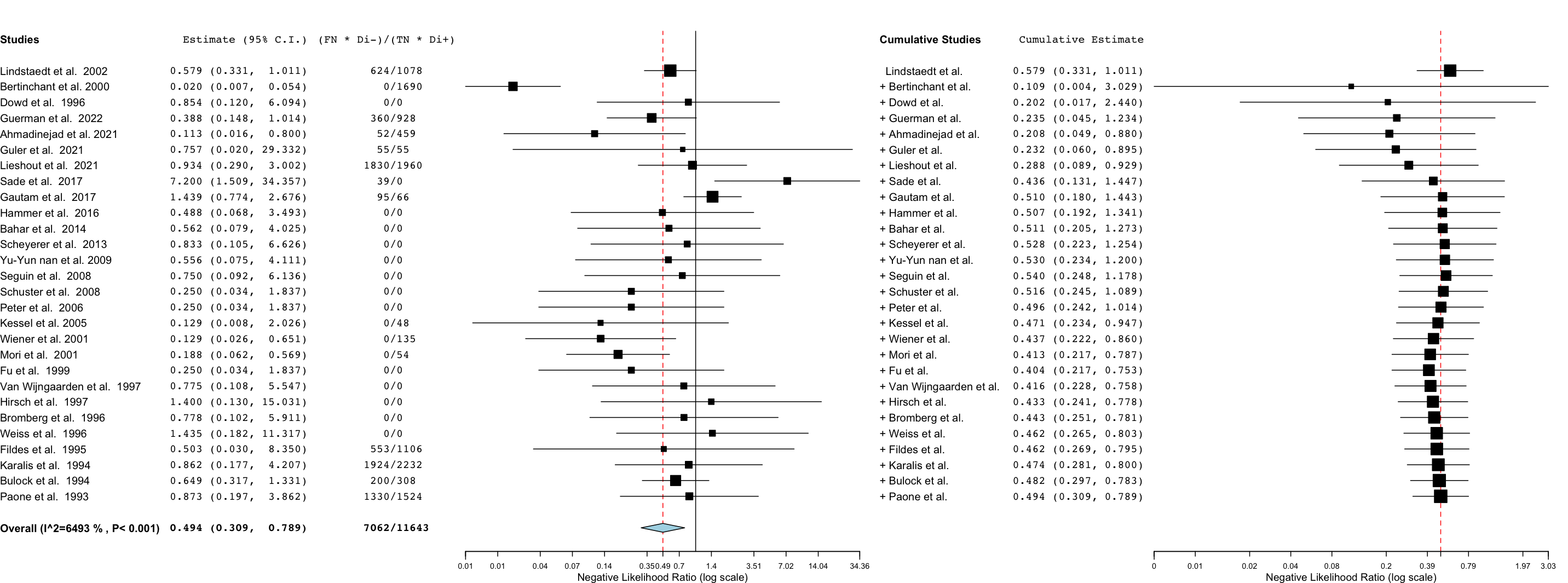

Supplement: Supplementary file 8 — Additional file 8. Supplementary Figure 7. ECG-NLR-Forest Plot. [file 13017_2023_504_MOESM8_ESM.png]

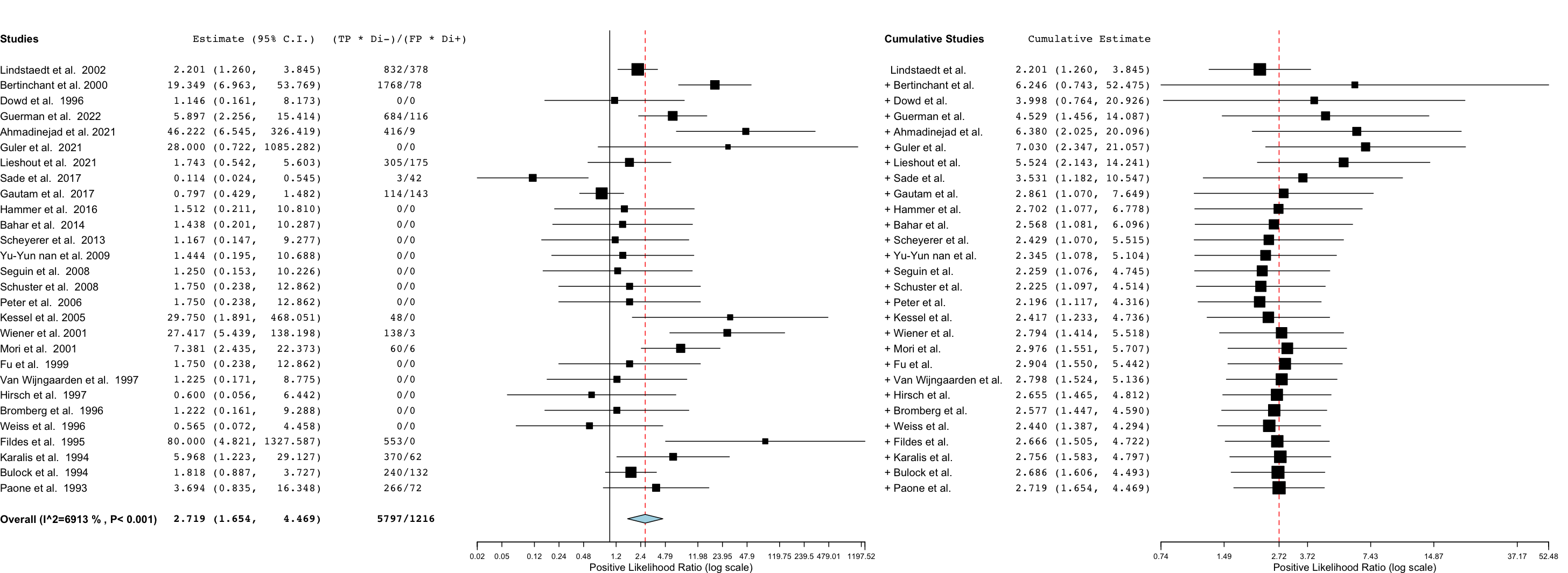

Supplement: Supplementary file 9 — Additional file 9. Supplementary Figure 8. ECG-PLR-Forest Plot. [file 13017_2023_504_MOESM9_ESM.png]

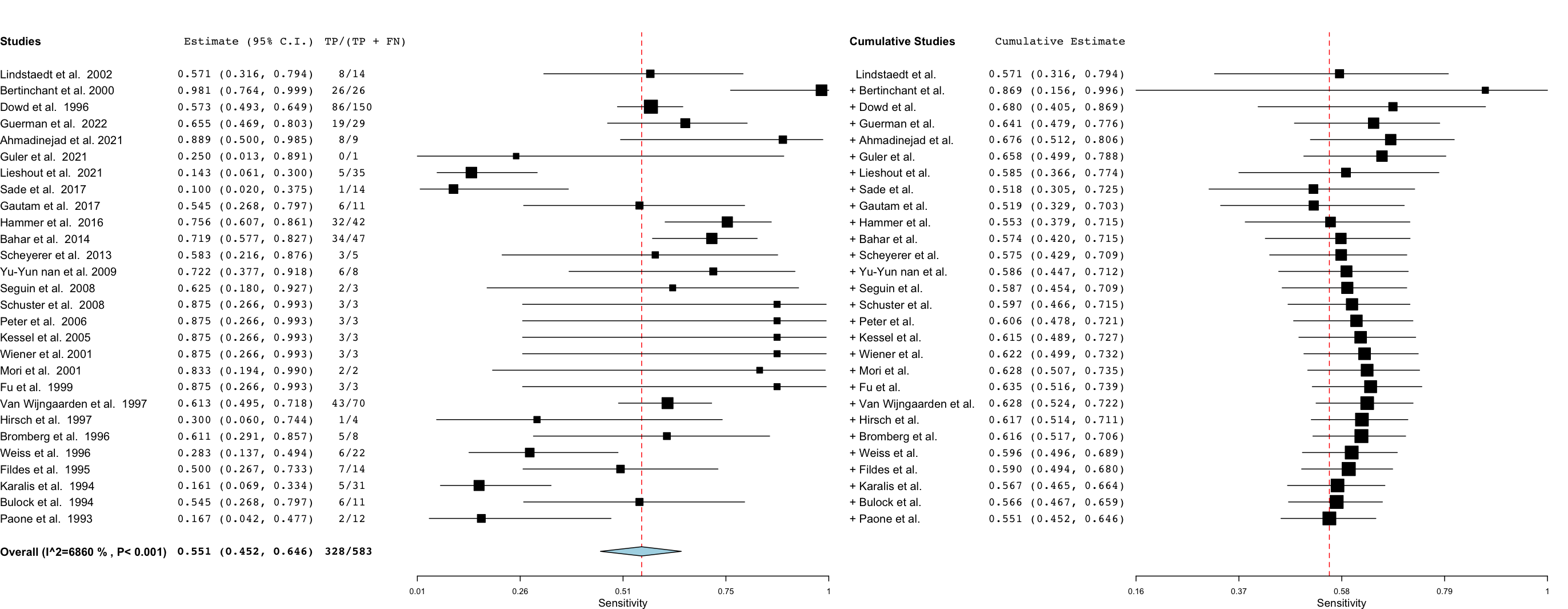

Supplement: Supplementary file 10 — Additional file 10. Supplementary Figure 9. ECG-Sens-Forest Plot. [file 13017_2023_504_MOESM10_ESM.png]

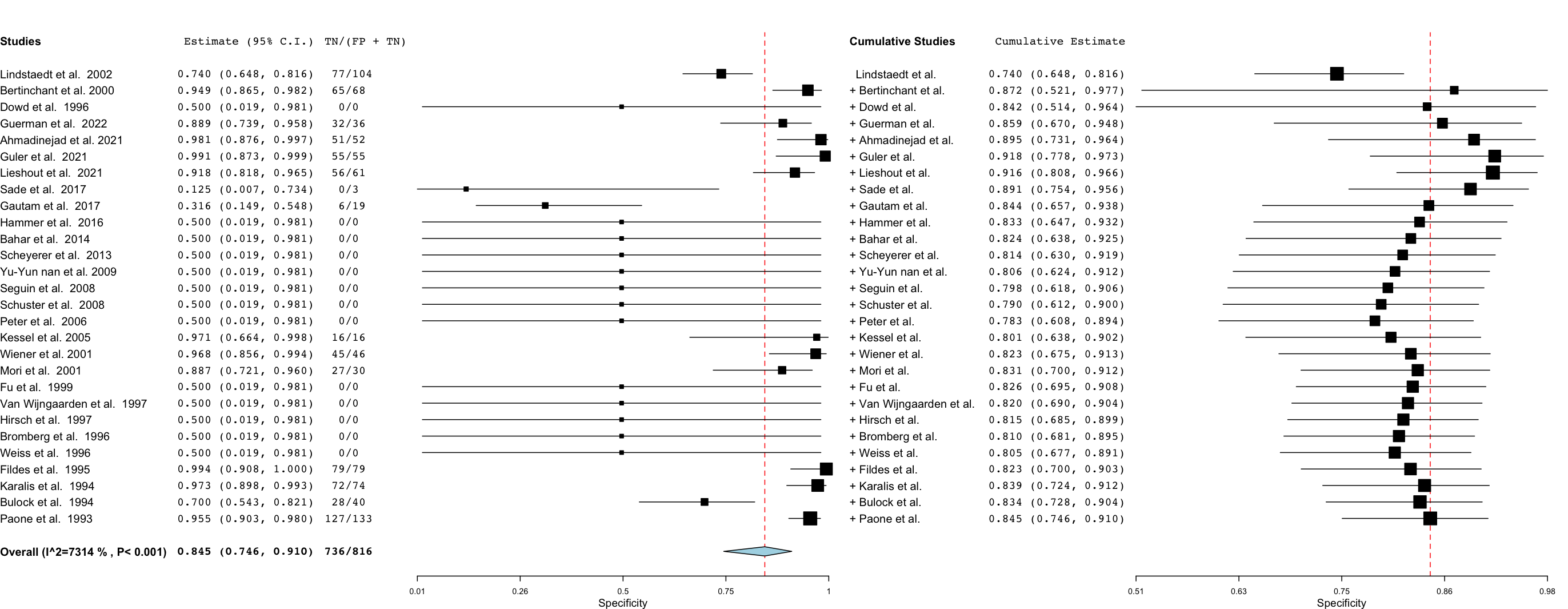

Supplement: Supplementary file 11 — Additional file 11. Supplementary Figure 10. ECG-Spec-Forest Plot. [file 13017_2023_504_MOESM11_ESM.png]

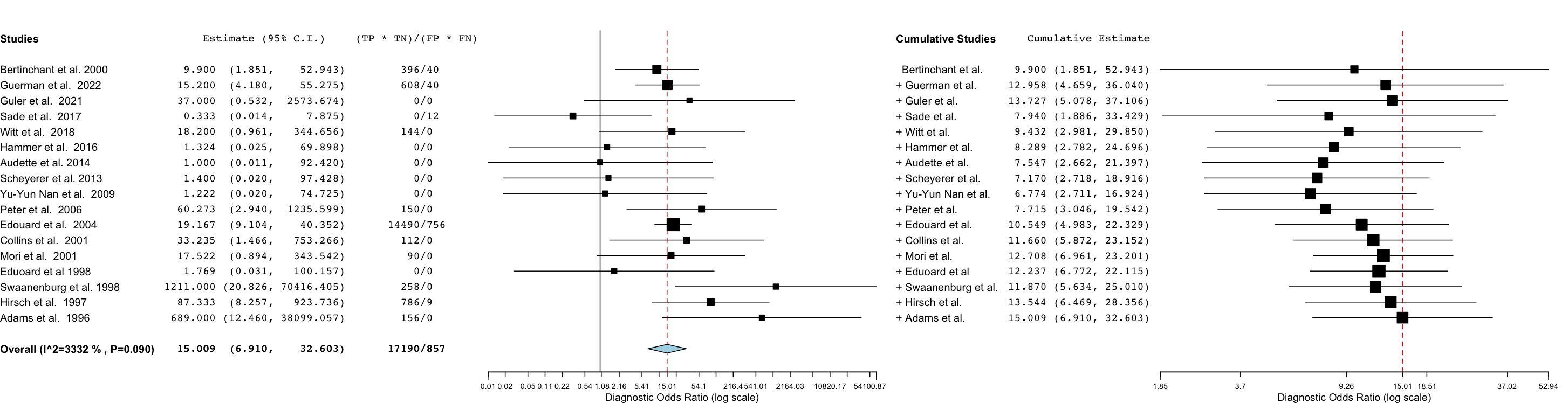

Supplement: Supplementary file 12 — Additional file 12. Supplementary Figure 11. TropI-DOR-Forest Plot. [file 13017_2023_504_MOESM12_ESM.png]

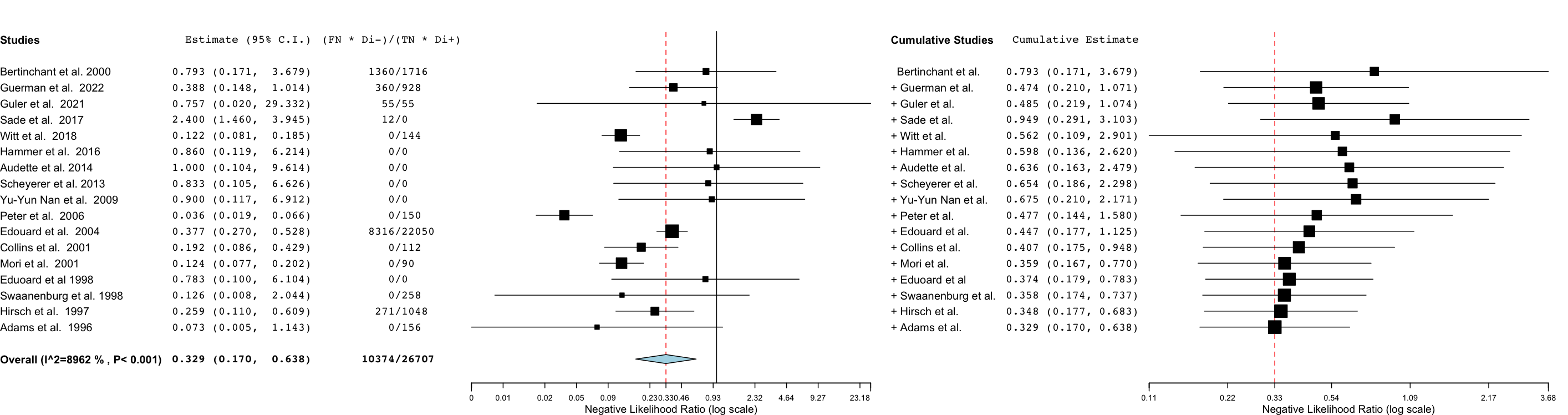

Supplement: Supplementary file 13 — Additional file 13. Supplementary Figure 12. TropI-NLR-Forest Plot. [file 13017_2023_504_MOESM13_ESM.png]

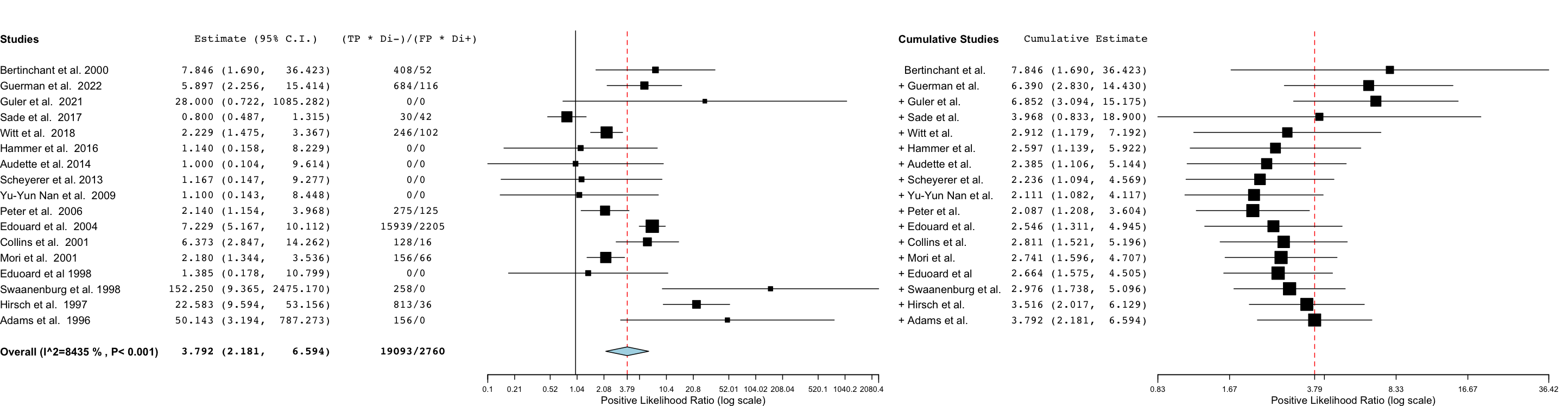

Supplement: Supplementary file 14 — Additional file 14. Supplementary Figure 13. TropI-PLR-Forest Plot. [file 13017_2023_504_MOESM14_ESM.png]

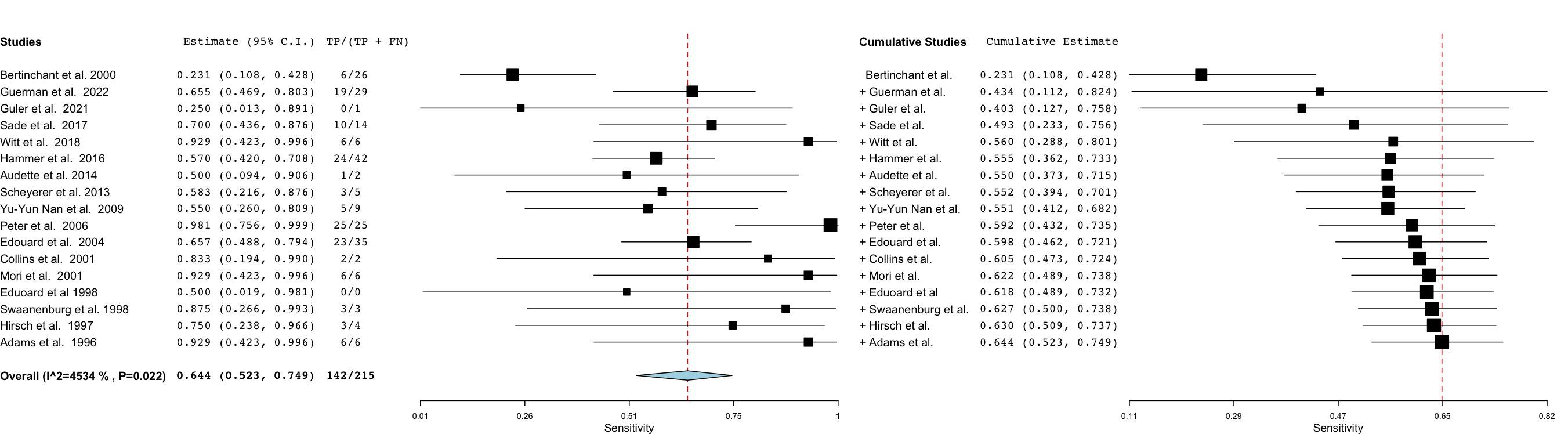

Supplement: Supplementary file 15 — Additional file 15. Supplementary Figure 14. TropI-Sens-Forest Plot. [file 13017_2023_504_MOESM15_ESM.png]

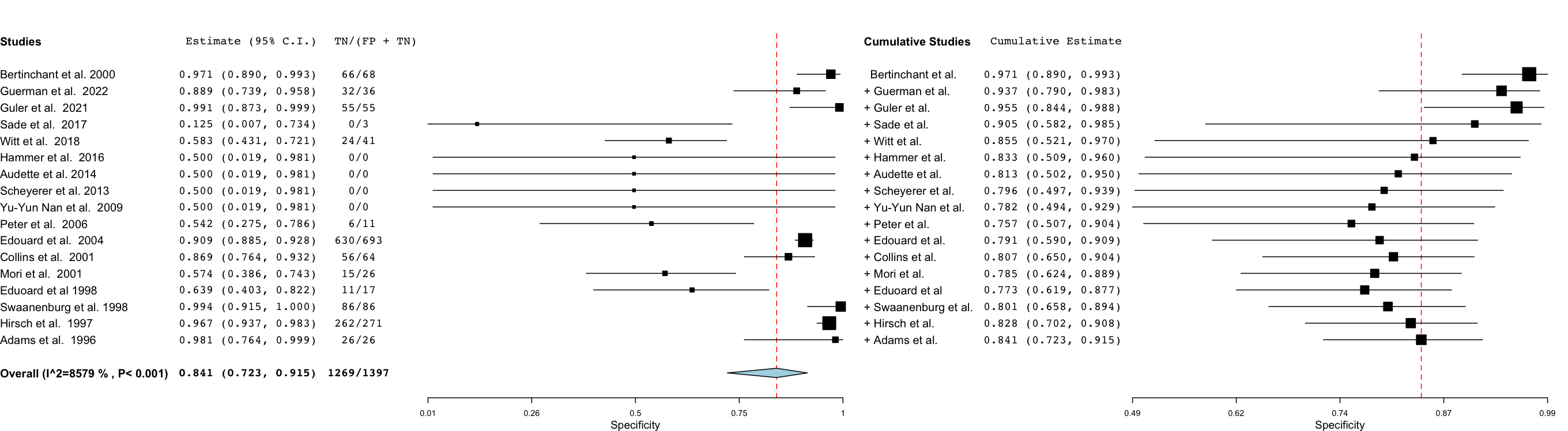

Supplement: Supplementary file 16 — Additional file 16. Supplementary Figure 15. TropI-Spec-Forest Plot. [file 13017_2023_504_MOESM16_ESM.png]

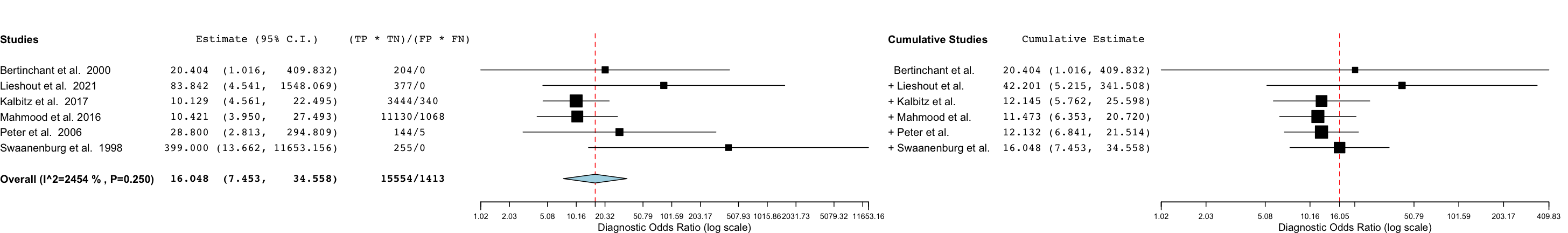

Supplement: Supplementary file 17 — Additional file 17. Supplementary Figure 16. TropT-DOR-Forest Plot. [file 13017_2023_504_MOESM17_ESM.png]

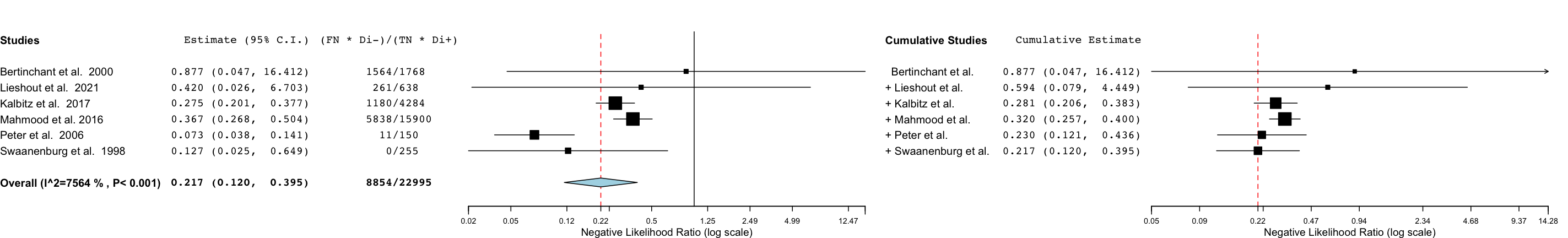

Supplement: Supplementary file 18 — Additional file 18. Supplementary Figure 17. Trop-NLR-Forest Plot. [file 13017_2023_504_MOESM18_ESM.png]

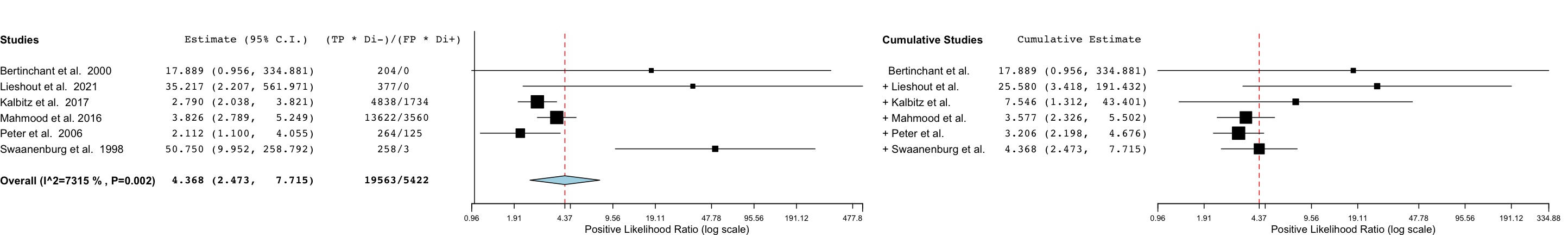

Supplement: Supplementary file 19 — Additional file 19. Supplementary Figure 18. TropT-PLR-Forest Plot. [file 13017_2023_504_MOESM19_ESM.png]

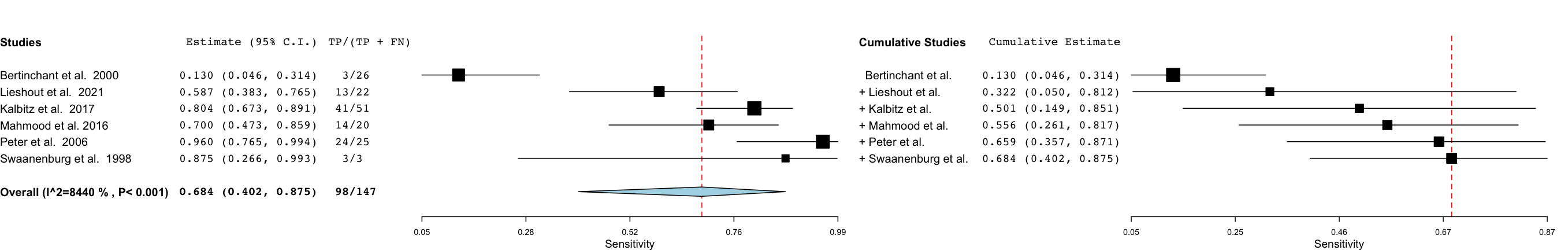

Supplement: Supplementary file 20 — Additional file 20. Supplementary Figure 19. TropT-Sens-Forest Plot. [file 13017_2023_504_MOESM20_ESM.png]

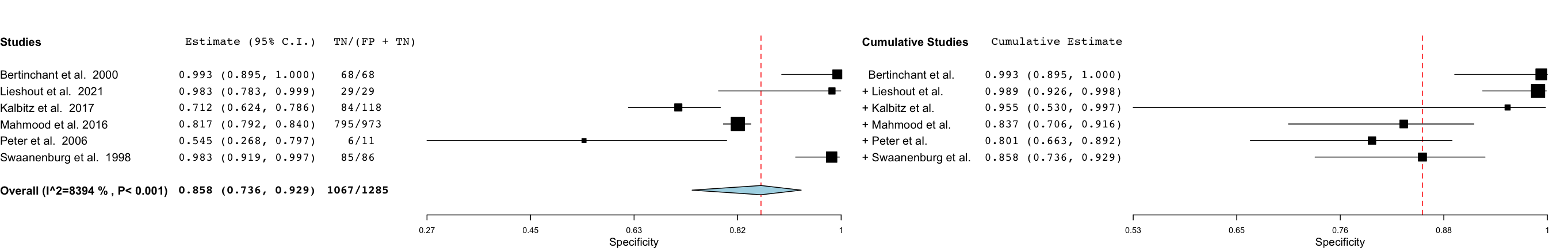

Supplement: Supplementary file 21 — Additional file 21. Supplementary Figure 20. TropT-Spec-Forest Plot. [file 13017_2023_504_MOESM21_ESM.png]

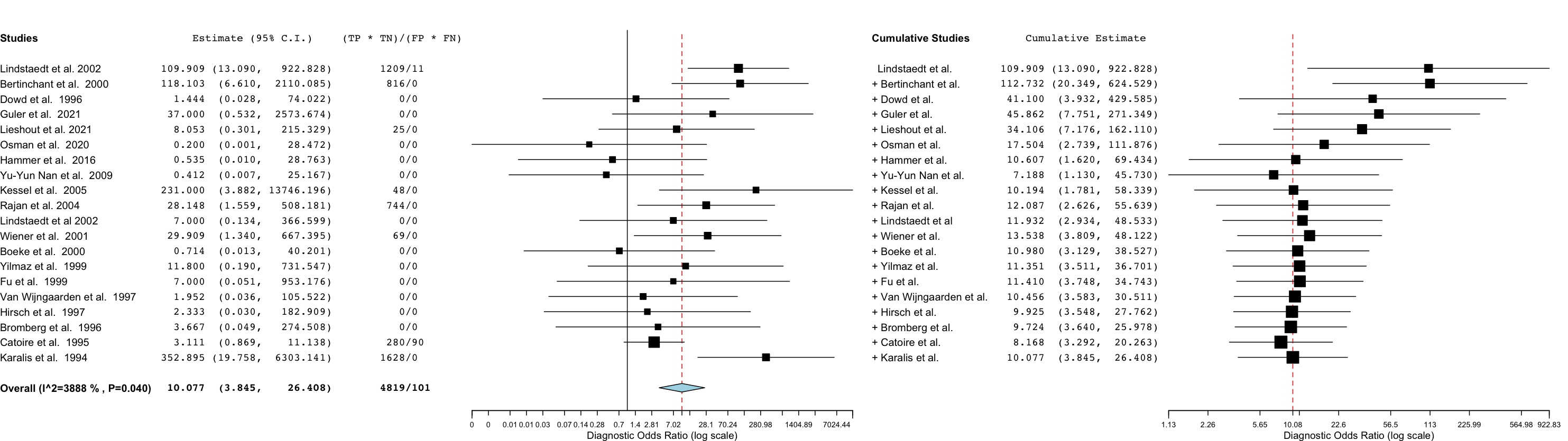

Supplement: Supplementary file 22 — Additional file 22. Supplementary Figure 21. TTE-DOR-Forest Plot. [file 13017_2023_504_MOESM22_ESM.png]

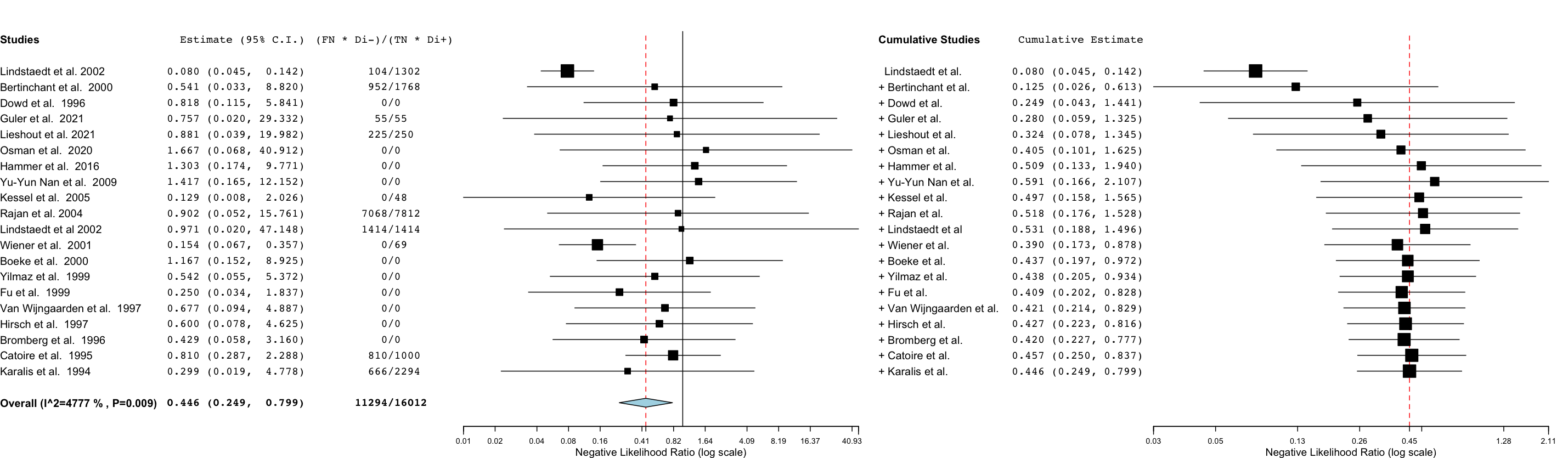

Supplement: Supplementary file 23 — Additional file 23. Supplementary Figure 22. TTE-NLR-Forest Plot. [file 13017_2023_504_MOESM23_ESM.png]

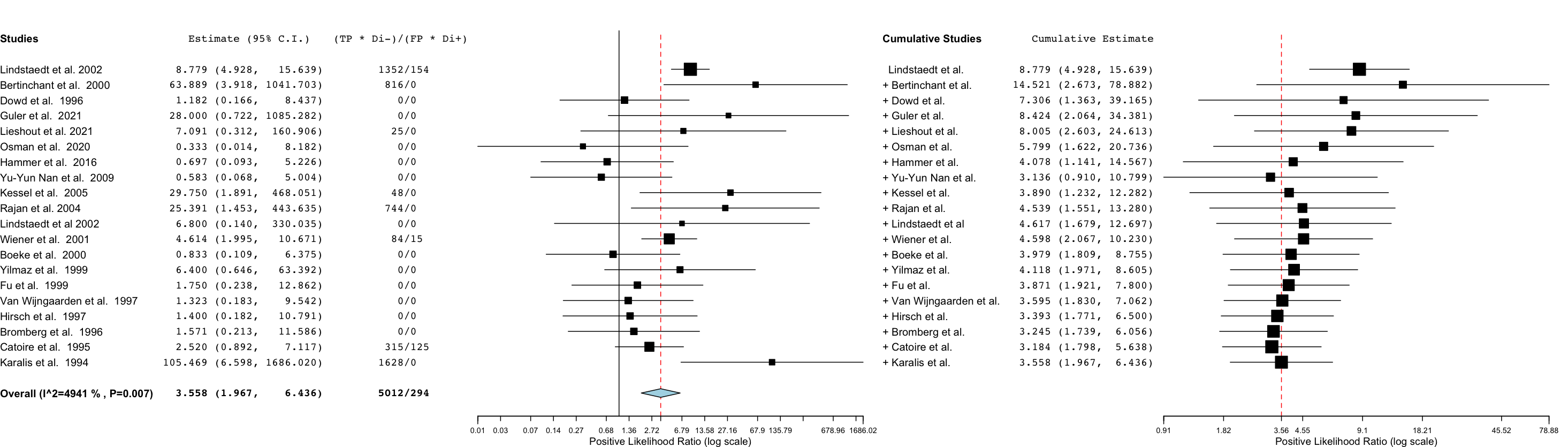

Supplement: Supplementary file 24 — Additional file 24. Supplementary Figure 23. TTE-PLR-Forest Plot. [file 13017_2023_504_MOESM24_ESM.png]

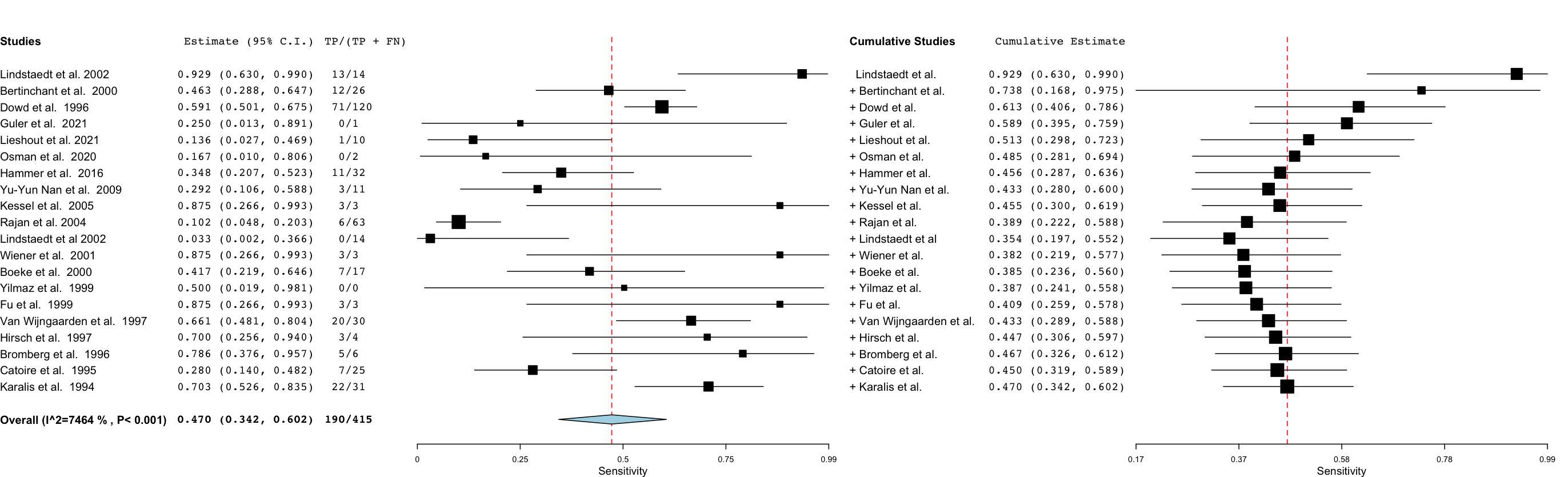

Supplement: Supplementary file 25 — Additional file 25. Supplementary Figure 24. TTE-Sens-Forest Plot. [file 13017_2023_504_MOESM25_ESM.png]

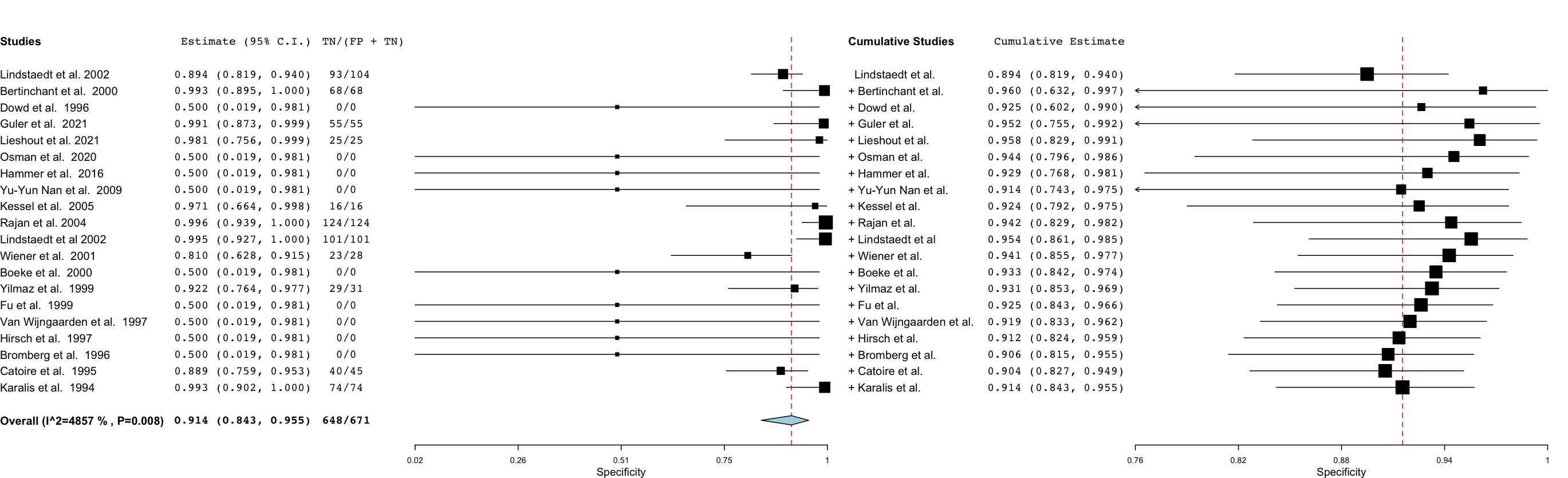

Supplement: Supplementary file 26 — Additional file 26. Supplementary Figure 25. TTE-Spec-Forest Plot. [file 13017_2023_504_MOESM26_ESM.png]

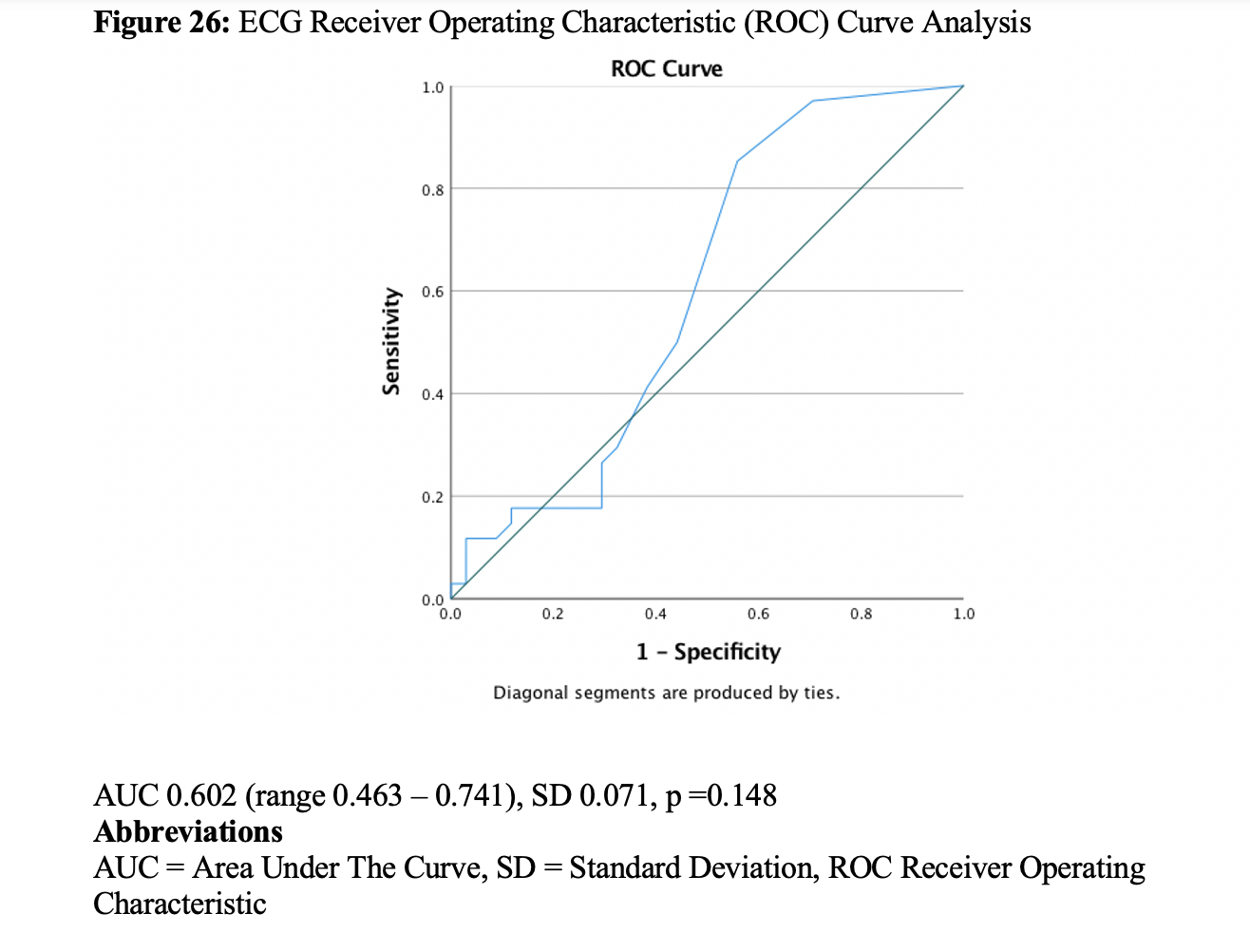

Supplement: Supplementary file 27 — Additional file 27. Supplementary Figure 26. ECG Receiver Operating Characteristic (ROC) Curve Analysis. [file 13017_2023_504_MOESM27_ESM.png]

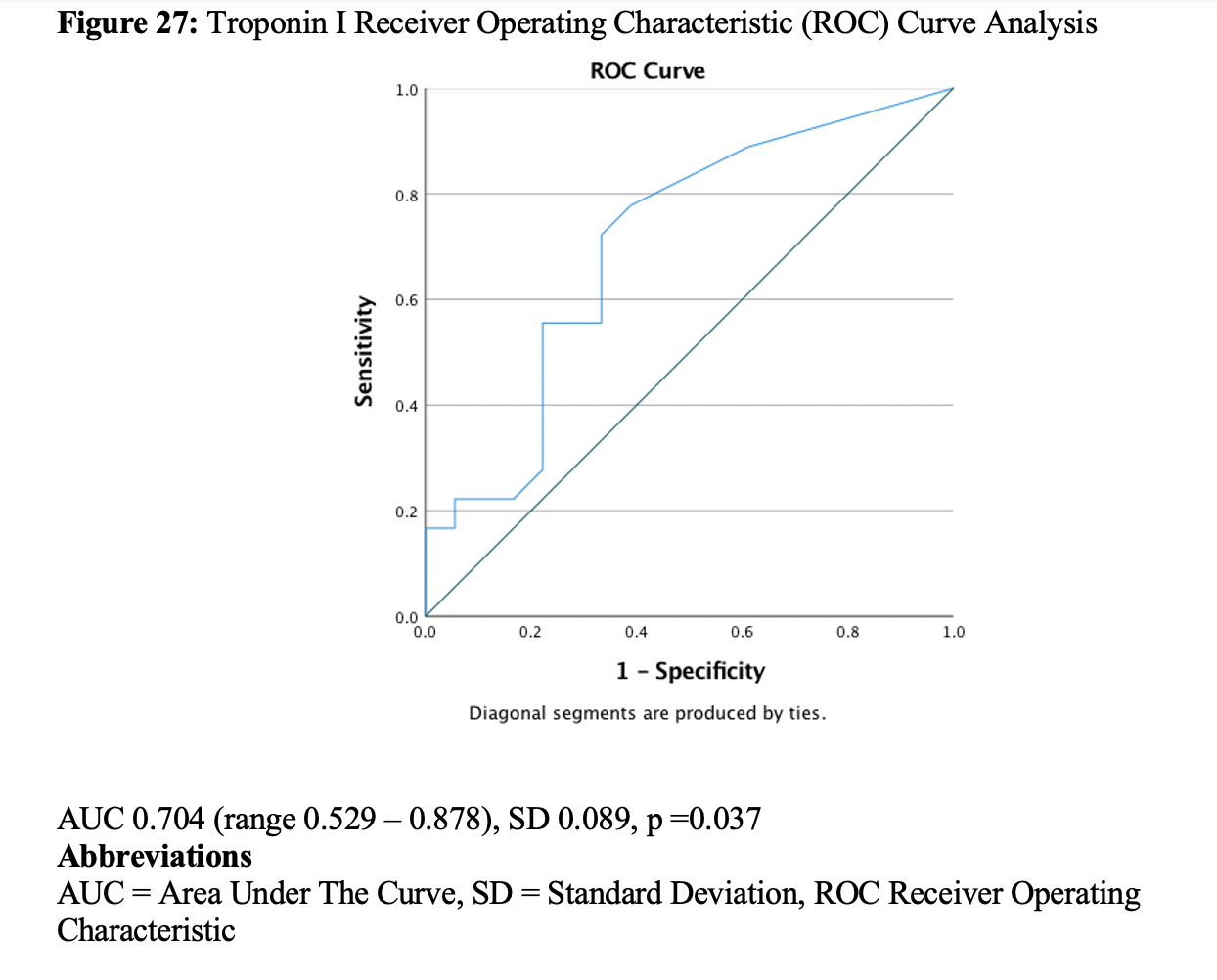

Supplement: Supplementary file 28 — Additional file 28. Supplementary Figure 27. Troponin I Receiver Operating Characteristic (ROC) Curve Analysis. [file 13017_2023_504_MOESM28_ESM.png]

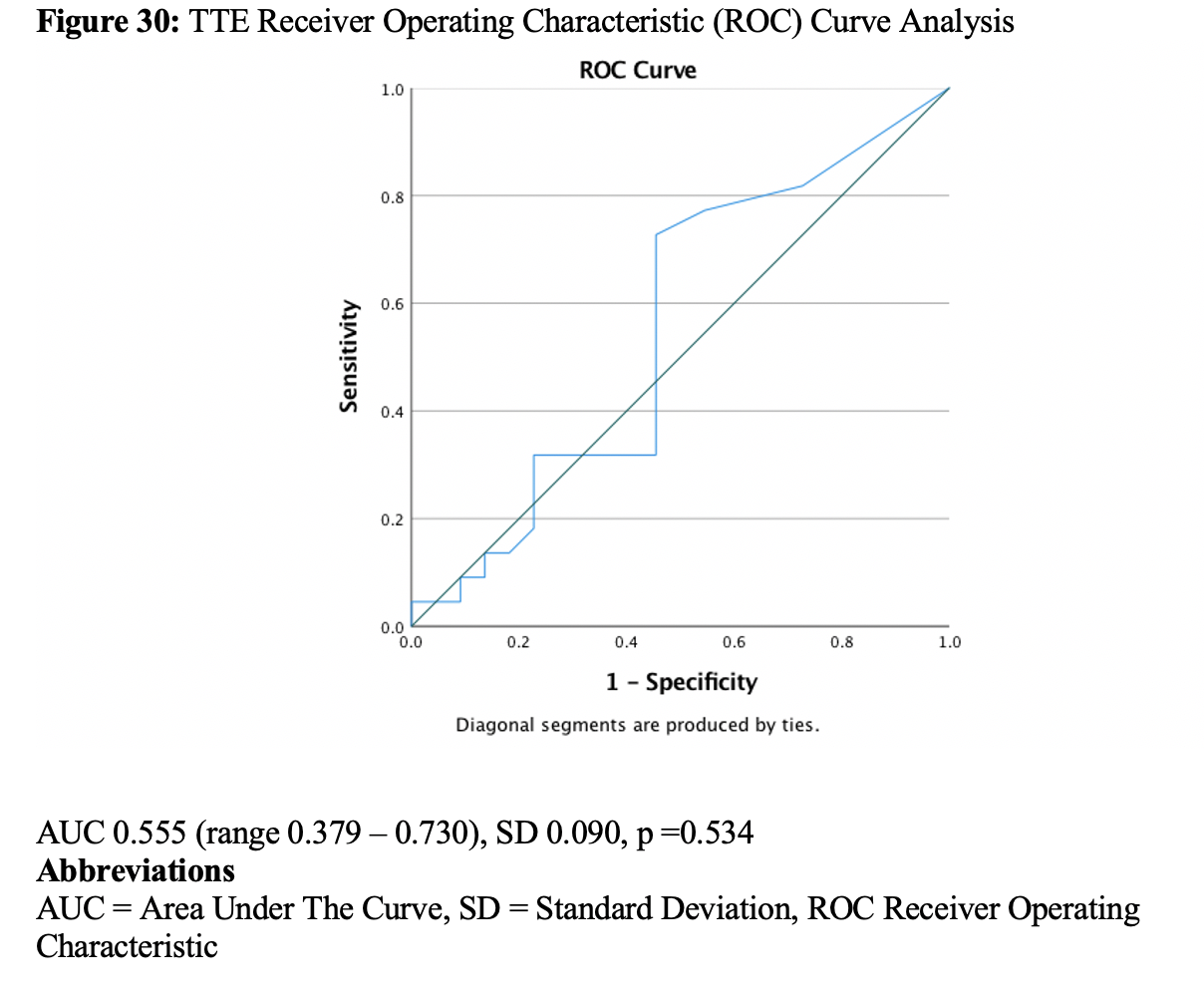

Supplement: Supplementary file 29 — Additional file 29. Supplementary Figure 28. TTE Receiver Operating Characteristic (ROC) Curve Analysis. [file 13017_2023_504_MOESM29_ESM.png]

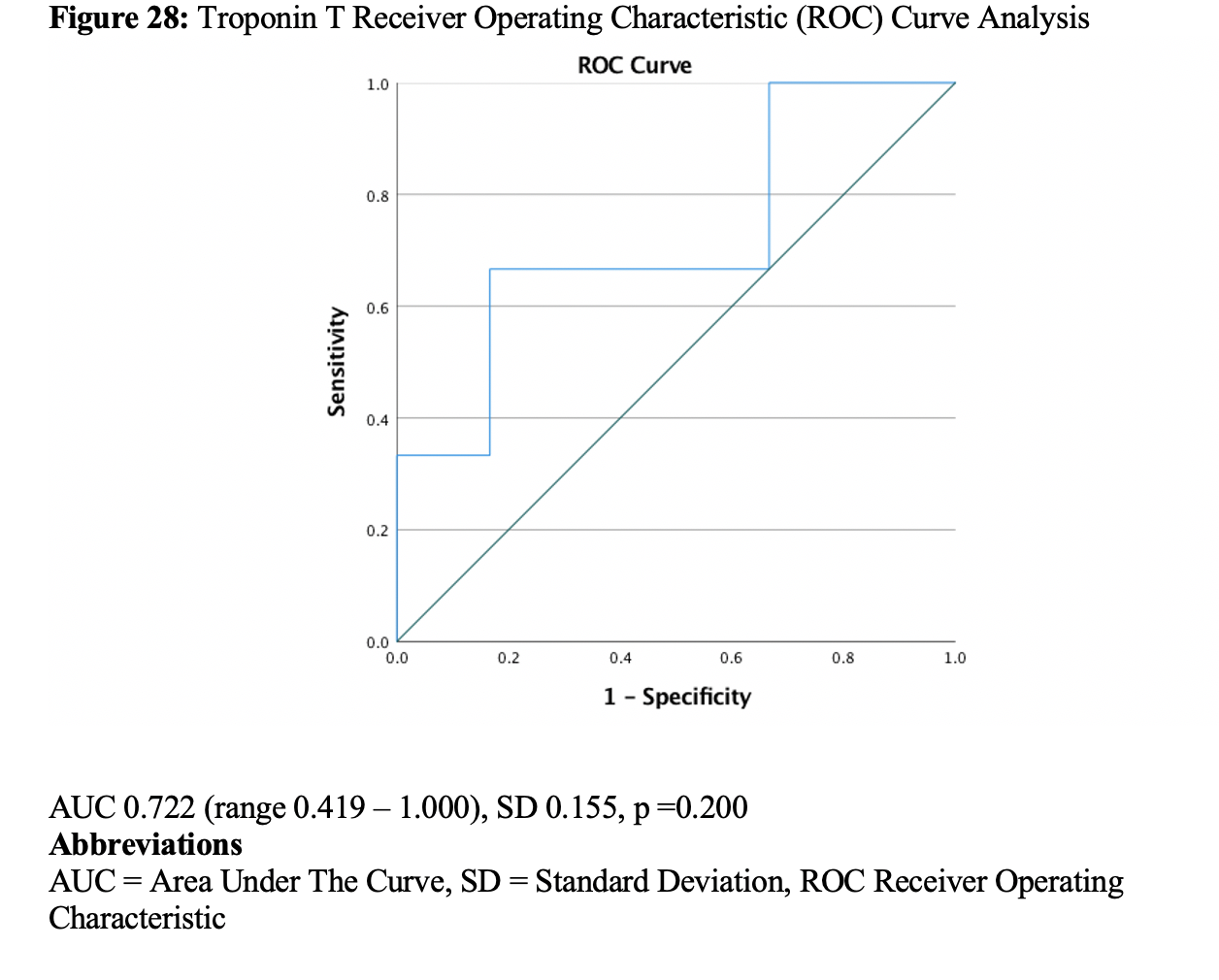

Supplement: Supplementary file 30 — Additional file 30. Supplementary Figure 29. Troponin T Receiver Operating Characteristic (ROC) Curve Analysis. [file 13017_2023_504_MOESM30_ESM.png]

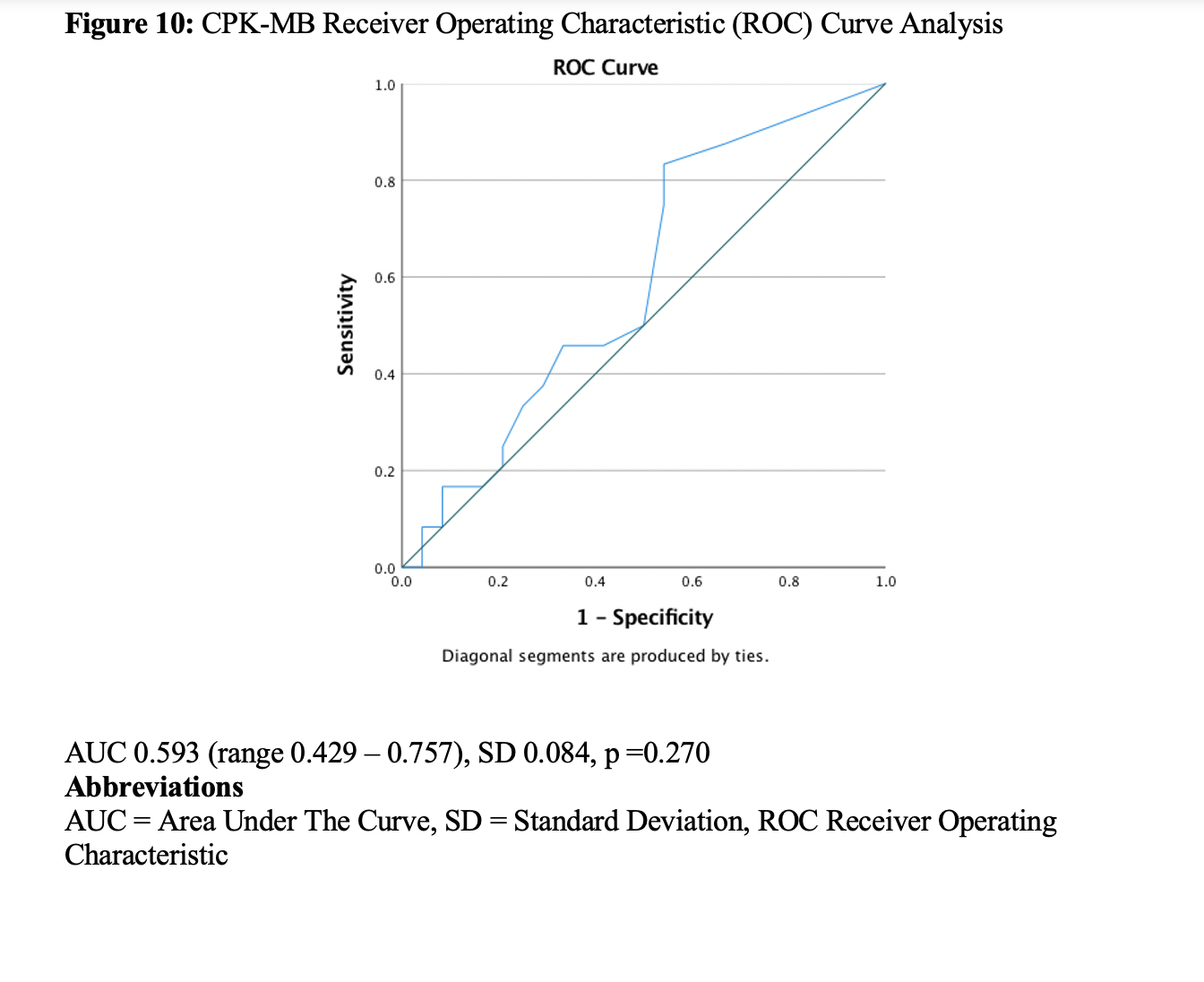

Supplement: Supplementary file 31 — Additional file 31. Supplementary Figure 30. CPK-MB Receiver Operating Characteristic (ROC) Curve Analysis. [file 13017_2023_504_MOESM31_ESM.png]
